# A Digital Tool for Clinical Evidence–Driven Guideline Development by Studying Properties of Trial Eligible and Ineligible Populations: Development and Usability Study

**DOI:** 10.2196/52385

**Published:** 2025-01-16

**Authors:** Shahzad Mumtaz, Megan McMinn, Christian Cole, Chuang Gao, Christopher Hall, Magalie Guignard-Duff, Huayi Huang, David A McAllister, Daniel R Morales, Emily Jefferson, Bruce Guthrie

**Affiliations:** 1 Health Informatics Centre School of Medicine University of Dundee Dundee United Kingdom; 2 Division of Population Health and Genomics School of Medicine University of Dundee Dundee United Kingdom; 3 School of Natural and Computing Sciences University of Aberdeen Aberdeen United Kingdom; 4 Advanced Care Research Centre Usher Institute The University of Edinburgh Edinburgh United Kingdom; 5 School of Health and Wellbeing University of Glasgow Glasgow United Kingdom; 6 Health Data Research UK London United Kingdom

**Keywords:** multimorbidity, clinical practice guideline, gout, Trusted Research Environment, National Institute for Health and Care Excellence, Scottish Intercollegiate Guidelines Network, clinical practice, development, efficacy, validity, epidemiological data, epidemiology, epidemiological, digital tool, tool, age, gender, ethnicity, mortality, feedback, availability

## Abstract

**Background:**

Clinical guideline development preferentially relies on evidence from randomized controlled trials (RCTs). RCTs are gold-standard methods to evaluate the efficacy of treatments with the highest internal validity but limited external validity, in the sense that their findings may not always be applicable to or generalizable to clinical populations or population characteristics. The external validity of RCTs for the clinical population is constrained by the lack of tailored epidemiological data analysis designed for this purpose due to data governance, consistency of disease or condition definitions, and reduplicated effort in analysis code.

**Objective:**

This study aims to develop a digital tool that characterizes the overall population and differences between clinical trial eligible and ineligible populations from the clinical populations of a disease or condition regarding demography (eg, age, gender, ethnicity), comorbidity, coprescription, hospitalization, and mortality. Currently, the process is complex, onerous, and time-consuming, whereas a real-time tool may be used to rapidly inform a guideline developer’s judgment about the applicability of evidence.

**Methods:**

The National Institute for Health and Care Excellence—particularly the gout guideline development group—and the Scottish Intercollegiate Guidelines Network guideline developers were consulted to gather their requirements and evidential data needs when developing guidelines. An R Shiny (R Foundation for Statistical Computing) tool was designed and developed using electronic primary health care data linked with hospitalization and mortality data built upon an optimized data architecture. Disclosure control mechanisms were built into the tool to ensure data confidentiality. The tool was deployed within a Trusted Research Environment, allowing only trusted preapproved researchers to conduct analysis.

**Results:**

The tool supports 128 chronic health conditions as index conditions and 161 conditions as comorbidities (33 in addition to the 128 index conditions). It enables 2 types of analyses via the graphic interface: overall population and stratified by user-defined eligibility criteria. The analyses produce an overview of statistical tables (eg, age, gender) of the index condition population and, within the overview groupings, produce details on, for example, electronic frailty index, comorbidities, and coprescriptions. The disclosure control mechanism is integral to the tool, limiting tabular counts to meet local governance needs. An exemplary result for gout as an index condition is presented to demonstrate the tool’s functionality. Guideline developers from the National Institute for Health and Care Excellence and the Scottish Intercollegiate Guidelines Network provided positive feedback on the tool.

**Conclusions:**

The tool is a proof-of-concept, and the user feedback has demonstrated that this is a step toward computer-interpretable guideline development. Using the digital tool can potentially improve evidence-driven guideline development through the availability of real-world data in real time.

## Introduction

Multimorbidity is the presence of 2 or more long-term health conditions in a person [1]. Typically, multimorbidity was considered a problem for older populations but is increasingly recognized as a challenge in younger people as well [2]. Multimorbidity is associated with worse physical and mental health function, higher service use, and higher mortality [3-5].

Health care services and research often focus on health conditions in isolation, but aging populations mean that both care and research need to better account for multimorbidity. A key point of the intersection of research evidence and care is clinical guideline development, which is where a systematic review of research evidence is used to derive recommendations for clinical practice.

Clinical guideline development preferentially relies on evidence from randomized controlled trials (RCTs). RCT is a gold-standard method to evaluate the efficacy of treatments because they have the highest internal validity [6]. However, the external validity of trial evidence may be limited in the sense that their findings may not always be applicable to or generalizable to clinical populations or population characteristics [7]. This is because RCTs commonly exclude large proportions of patients treated in normal practice. In a systematic review of eligibility for 305 RCTs of treatments for physical conditions, half of the trials excluded more than three-quarters of people with the condition, most commonly older people and people with significant comorbidity (conditions apart from the target of the trial treatment) or coprescribing (of treatments for other conditions) [6,8].

The problem of exclusion from trials is commonly recognized but less commonly quantified. Systematic review guidelines state that generalizability should always be considered and discussed, but rarely is, even in Cochrane systematic reviews [9,10]. The Grades of Recommendation Assessment, Development, and Evaluation (GRADE) recommendations for guideline development also include a method for considering the “directness” of evidence [7]. In principle, guideline developers following GRADE [11] already consider applicability as part of the assessment of indirectness. However, judgments about applicability are often implicit, and the generalizability of estimates (eg, of intervention effect) from RCT evidence is more often assumed than explicitly examined. In practice, clinical guidelines rarely refer to comorbidity or its implications [12], and guidelines rarely mention treatment interactions even though following single-condition guideline recommendations for treatment in a person with multiple conditions commonly leads to potentially serious drug-disease and drug-drug interactions [13], and the cumulative impact of individually rational guideline treatment recommendations can easily be irrational or contradictory in people with multimorbidity [13-17].

Better accounting for multimorbidity and polypharmacy in single-condition guidelines requires robust judgments about the applicability and generalizability of RCT evidence. Such judgments have to be informed by an epidemiological understanding of the clinical population and how it compares to the population studied in RCTs. However, guideline developers are constrained by the lack of tailored epidemiological data analysis designed for this purpose due to data governance, consistency of disease definitions, and reduplicated effort in analysis code. Here, we present a proof-of-concept digital tool using UK health care data to systematically characterize the index condition population, supporting the reuse of existing disease definitions and analysis and integrated disclosure control to ensure governance requirements. A key motivation was to develop an efficient tool that did not require expert data manipulation or technical skills.

## Methods

### Study Design

We worked with domain experts in guideline development (groups from the National Institute for Health and Care Excellence [NICE] and the Scottish Intercollegiate Guidelines Network [SIGN]) to understand what clinical evidence would be useful for the epidemiological understanding of the disease in terms of overall population and clinical trial eligible or ineligible populations. The Clinical Practice Research Datalink (CPRD) GOLD [18] was used as a primary care data source. The CPRD GOLD is a structured data set extracted from primary care electronic health records (INPS Vision software; In Practice Systems Limited) in the United Kingdom, where key data quality standards are met [19]. In the following subsections, an overview is provided of the data set, details of the requirements gathering process, the design of the underpinning data structure to support real-time querying, the development of the software tool, deployment of the tool within a Trusted Research Environment (TRE; a secure data and analytics environment), the ethical approval process, and an evaluation of the tool.

### Data Set (Primary Care Data Linked with Hospitalization and Mortality)

Data were extracted from the CPRD GOLD data set, consisting of primary care data from general practice electronic health records (EHRs) linked to administrative data for hospital admissions and mortality registration—the organization that manages the CPRD GOLD data extract provided linked data [20]. Data included all permanently registered patients at participating practices on November 30, 2015 (1.17 million), with at least 2 years of prior registration. Clinical conditions were measured any time before the index date, while data on prescriptions were restricted to the previous year due to the size of the data files and clinical relevance for guideline development. Follow-up data for hospital admission and death were available for 3 years (ie, until November 30, 2018). The presence of 161 long-term conditions was ascertained using Read codes and ICD10 (International Classification of Diseases 10th Revision) codes recorded on or before November 30, 2015, using published code lists from the Health Data Research UK phenotype library [21,22]. Read codes and ICD10 coding systems have been used for coding patient data across the UK National Health Services in primary and secondary care services, respectively. For each patient, the electronic frailty index (eFI) [23] and the Charlson Comorbidity Index score [24] were calculated. The raw data set used for this study was provided by the organization that manages CPRD, which explained the data quality and validation checks in place [25]. For study-specific cohort quality and validity, the first and second authors of this paper conducted separate analyses for several conditions, and the cohort sizes generated through the tool and analysis scripts were observed to be the same.

### Requirements Gathering (Live Guideline Development Group Consultation)

Tool development was done in conjunction with the guideline development group (GDG) for the NICE gout guideline (subsequently referred to as the NICE gout GDG); therefore, gout was used as an exemplar index condition. Gout is a form of arthritis caused by urate crystal deposition in joints that causes severe joint inflammation and pain, which can be prevented by long-term medication to reduce blood urate levels [26,27]. Initial interviews with GDG were used to define requirements and active engagement with the NICE gout GDG throughout its life cycle, including providing bespoke analysis upon request to help prompt reflection on the use of different kinds of data at different points in guideline development. The bespoke analysis provided is published as an appendix to the guideline [28]. In parallel, a prototype data analysis tool was designed and demonstrated to the NICE gout GDG and other guideline developers in NICE and SIGN to seek their feedback and more detailed requirements in terms of data analysis they wished to have access to as they considered the evidence and drafted recommendations. It was agreed to capture the following two high-level output types:

1. Index condition population analysis: A general view of the population with the underlying condition.
2. Clinical trial population analysis: A modified view allowing explicit comparison of patients eligible or ineligible for a particular clinical trial (or a notional clinical trial), where the user inputs inclusion or exclusion criteria to define the 2 populations: eligible versus ineligible.

Both types of analysis were agreed to produce summaries as tables of numbers for age, gender, ethnicity, and indices of multiple deprivations (IMD) groupings for the selected condition. These summaries were also expanded to demonstrate frailty (measured by the eFI), comorbidity (measured with the Charlson score), the prevalence of comorbidities, and current drug prescription. Additionally, it was highlighted to produce rates of hospitalization and mortality.

Four further requirements included: (1) making the analysis pipeline available for other chronic conditions (because of perceived value across different health conditions by GDGs); (2) supporting the selection of hierarchical groupings of comorbidities and drug prescriptions; (3) statistically disclosure controlled outputs to make egressing of the outputs faster (for instance, by rounding all numbers to nearest 10 and percentages to discrete numbers) to ensure patient confidentiality and to satisfy data governance approvals; and (4) generation of printable reports to use as evidence within the guideline development process, and for subsequent publication as part of the guideline.

### Optimizing the Data Structure

A major challenge when developing digital tools that use large data sets is the query processing time. For example, the CPRD GOLD data set has approximately 1.17 million patients in the patient file and approximately 254 million entries in the clinical table (storing codes documenting interactions with the practice). Extracting comorbidities for an index condition based on age, gender, and other features required intensive computation power and storage capacity when dynamic linking the patient and clinical tables. The same challenge was seen for other data analyses. To optimize query processing time, we designed new tables in the Microsoft SQL server, which included only the necessary entries for analysis (eg, for the presence of a condition, then only the first Read code recording its presence and the date recorded is required, rather than all such codes). Similarly, eFI and the Charlson scores were precalculated. However, even with an optimized data structure, the performance of processing queries will increase given the cohort size for a selected index condition unless we increase the computing power and memory. The details of the new data structure design and the rationale for generating each table are given in Multimedia Appendix 1.

### Development of the Informatics Tool Using R Shiny

The study has developed an R Shiny (R Foundation for Statistical Computing) proof-of-concept digital informatics tool. R Shiny uses R packages to build graphical, interactive dashboards for data exploration [29]. Dashboards can be rapidly configured, making R Shiny an ideal tool for prototyping.

The tool dashboard design has considered the novices to analyze and extract useful information without knowledge of writing analysis scripts in R. The dashboard enables analysis of all patients with a chosen index condition (for instance, everyone with gout on November 30, 2015) and clinical trial analysis in terms of eligible and ineligible populations as defined in the requirements gathering process. Further descriptions of the analysis types with input and output of each type are presented in Multimedia Appendix 2.

### Comorbidities and Prescriptions Analyses

A key requirement of the tool output was to support a selection of comorbidities and drug prescriptions at different hierarchical grouping levels for the selected index condition of interest. The hierarchical groupings of conditions for comorbidities analysis were determined by 2 expert clinicians (authors) and are provided in Multimedia Appendix 3, whereas the British National Formulary (BNF) [30] was used to hierarchically group drugs. Diseases are classified into 3 hierarchical levels: body system, condition group, and individual conditions. The body system is the top level in the hierarchy of diseases. Further down the hierarchy are condition groups, which are subcategories of the body system and are made up of individual conditions. For example, the “cancers” body system has condition groups such as “hematological cancers,” “solid organ cancer (primary),” or “solid organ cancer (secondary),” and “hematological cancer” is a condition group made up of individual conditions such as “Hodgkin lymphoma,” “leukemia,” “myelodysplastic syndrome,” “non-Hodgkin lymphoma,” “plasma cell malignancy,” and “polycythemia vera.”

When presenting comorbidities at the body system level, if a patient has one or more comorbidities, under the hierarchical subcategories of the body system, they will be counted as 1 disease or condition. The tool’s design considers a selection of any level of comorbidities in the disease’s hierarchy.

For prescription data within CPRD GOLD, drug codes are given as BNF codes so that drug classification was used instead of the World Health Organization (WHO) Anatomical Therapeutic Chemical classification, which is the de facto drug coding standard in clinical trials [31]. In the BNF, drugs are classified into 3 hierarchical levels: drug chapters, drug classes, and individual drugs.

### Deployment of the R Shiny Tool

**Figure 1:**
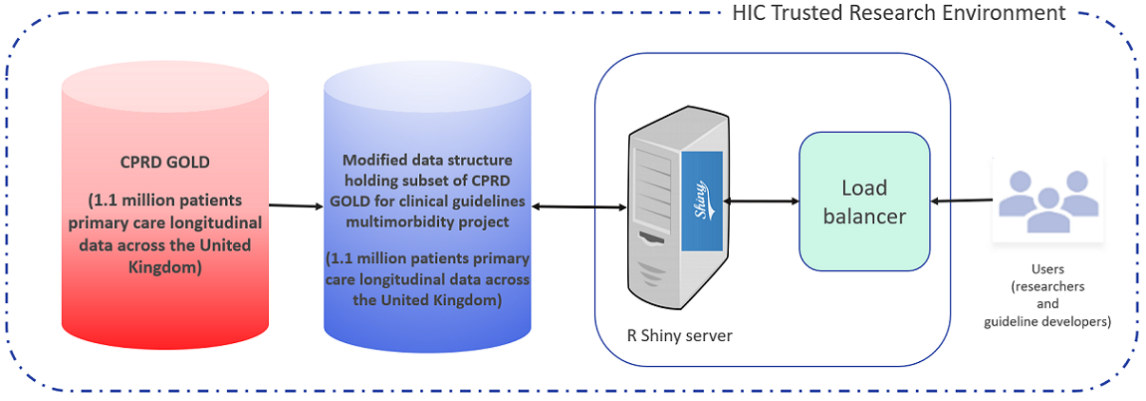
A schematic view of the deployed R Shiny tool. A load balancer allows multiple users to interact simultaneously with an R Shiny server. CPRD: Clinical Practice Research Datalink; HIC: Health Informatics Centre.

The R Shiny tool was developed and deployed within the Health Informatics Centre (HIC) of the University of Dundee TRE, which was only accessible to authorized members of the study (Figure 1). The tool is an R Shiny application, and it can be accessed simply through a web browser within the TRE. The tool applies disclosure controls to the results displayed by rounding all numbers to the nearest 10 and providing percentages as discrete numbers, thereby avoiding accidental patient reidentification through inference without introducing bias in prevalence estimates. All data presented here fulfills the disclosure control requirements.

### Ethical Considerations

The study only used deidentified data, with integrated disclosure control within the tool to avoid accidental patient reidentification through inference, and was approved by the CPRD Independent Scientific Advisory Committee (ISAC; protocol 20_018) [32]. To ensure patient privacy, CPRD provided anonymized data for this study.

### Evaluation of the Tool

**Figure 2:**
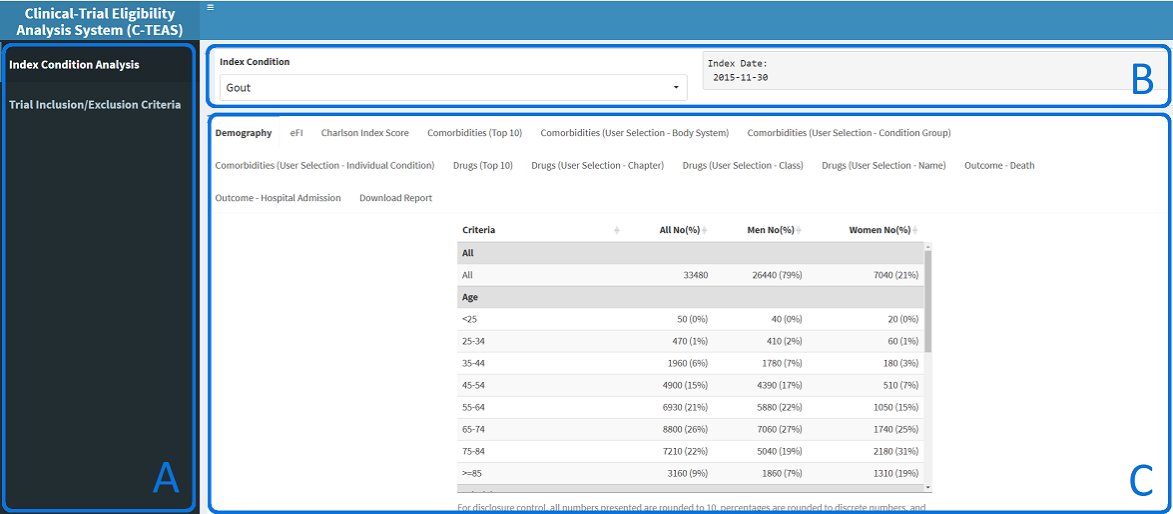
A screenshot of the R Shiny tool for clinical population analysis. The left sidebar (A) has 2 menu items: index condition population analysis and trial eligible or ineligible population analysis. The top section (B) enables users to choose an index condition of interest from the drop-down list. The bottom right section (C) shows the results of the selected index condition in different tabs focusing on different outputs such as demographic distributions, comorbidities, and coprescriptions.

Throughout the development of the tool, we performed iterative usability testing by demonstrating updated versions to the domain experts to confirm the applicability of the evidence. The domain experts included gout GDG, the study advisory group (which included representatives from NICE and SIGN), the European Respiratory Society, and public contributors. For the final evaluation, the project team members and external users from NICE and SIGN were given controlled access to trial the tool within the HIC TRE. Simple three-step instructions were shared with users: (1) logging into the HIC TRE virtual machine, (2) typing a given URL in a browser to access the tool, and (3) conducting analysis using the GUI (Figure 2) as described in requirements gathering section earlier.

## Results

### Overview

During the analysis phase of the index conditions of interest, we considered factors in designing and developing an R Shiny tool that can be widely applicable to many conditions in CPRD GOLD. The tool supports the selection of 128 conditions as index conditions and 161 conditions as comorbidities (the 128 index conditions plus 33 other chronic health conditions), which are individual conditions in the hierarchical groupings and the associated higher-level groupings in the hierarchy (details of the supported index conditions and comorbidities are presented in Multimedia Appendix 3). CPRD is broadly representative of the UK population [33], and data quality is high, including gout [34].

### Overview of R Shiny Tool

**Figure 3:**
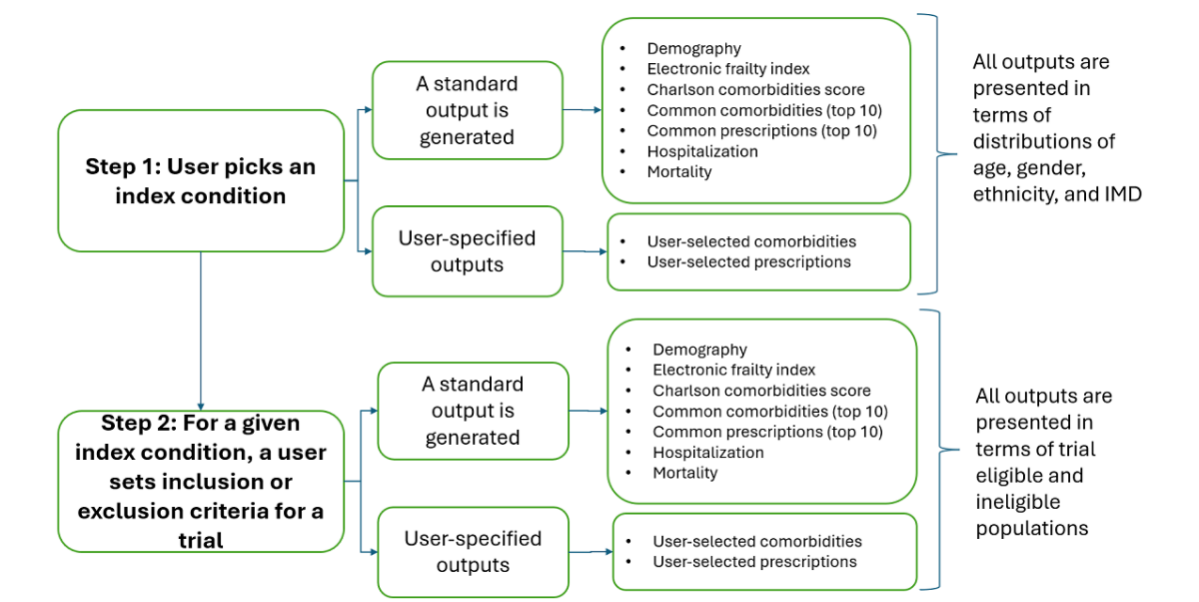
A workflow of an R Shiny tool. IMD: index of multiple deprivations.

The R Shiny tool supports a 2-step analysis process as described in the methodology section (Figure 3). First, a user performs population-level analysis for the condition of interest from the list of supported index conditions (Multimedia Appendix 3). Second, a user sets the trial inclusion and exclusion criteria to understand trial eligible or ineligible populations. A screenshot of the R Shiny tool is presented in Figure 2, highlighting three areas: (1) Figure 2A has 2 menu options that allow users to switch between analysis types; (2) Figure 2B is the area that allows the user to select the condition of interest from the drop-down list to generate population analysis; and (3) Figure 2C is the results area where multiple tabs are provided to enable switching between different types of outputs.

### Index Condition Population Analysis in Gout

The tool was designed to be flexible and provide many selection options to the end user to support guideline development; given the many combinations of results with 13 output tabs available, only a few examples are shown here.

We present results for gout as an index condition for demonstration purposes. Table 1 shows an analysis of the whole population with gout: any patient diagnosed with gout on or before the index date (November 30, 2015) is included in the analysis. Details are provided for distributions of patients’ characteristics, including age, gender, ethnicity, IMD, and time since diagnosis (prevalent and recently incident populations) at the index date. From a population of 1,168,620 patients from the CPRD GOLD data set, a cohort with gout of 33,480 (2.86%) was identified. The cohort with gout was proportionately older (aged greater than 64 years; 75%). The CPRD GOLD data set is mostly a White ethnic population (68%) and a relatively higher percentage (81%) is observed in the population with gout. For other ethnicity groupings: South Asian (3% vs 2%), Black (2% vs 1%), Chinese or Mixed or Other (2% vs 1%), missing (24% vs 15%). Based upon IMD, a higher percentage of those with gout were richer (26% in the least deprived category) versus poorer (13% in the most deprived category). For many diseases, the opposite trend is observed; however, gout has historically been known as “a rich man’s disease” [35-37], a generalization that is evidenced by this data.

**Table 1:** An example output of population with gout from the R Shiny application describing the distribution of patients’ characteristics, including age, gender, ethnicity, IMD^a^, and time since diagnosis (prevalent and recently incident populations).^b^

Criteria (feature)			All, n (%)		Men, n (%)		Women, n (%)
All			33,480 (100)		26,440 (79)		7040 (21)
**Age (years)**							
	Less than 25	50 (0)		40 (0)		20 (0)	
	25-34	470 (1)		410 (2)		60 (1)	
	35-44	1960 (6)		1780 (7)		180 (3)	
	45-54	4900 (15)		4390 (17)		510 (7)	
	55-64	6930 (21)		5880 (22)		1050 (15)	
	65-74	8800 (26)		7060 (27)		1740 (25)	
	75-84	7210 (22)		5040 (19)		2180 (31)	
	85 or older	3160 (9)		1860 (7)		1310 (19)	
**Ethnicity**							
	Black	320 (1)		240 (1)		80 (1)	
	Chinese or Mixed or Other	360 (1)		290 (1)		70 (1)	
	South Asian	610 (2)		470 (2)		140 (2)	
	White	27100 (81)		20860 (79)		6250 (89)	
	Missing	5090 (15)		4590 (17)		500 (7)	
**IMD quintile**							
	Q1 (least deprived)	8720 (26)		7130 (27)		1590 (23)	
	Q2	7400 (22)		5890 (22)		1510 (21)	
	Q3	7300 (22)		5710 (22)		1580 (22)	
	Q4	5680 (17)		4330 (16)		1350 (19)	
	Q5 (most deprived)	4380 (13)		3380 (13)		1010 (14)	
**Time since diagnosis (years)**							
	1 or greater	31,210 (93)		24,790 (94)		6420 (91)	
	Less than 1	2270 (7)		1650 (6)		620 (9)	

^a^IMD: index of multiple deprivations.

^b^For disclosure control, all numbers are rounded to 10, percentages are rounded to discrete numbers, and percentages presented are column percentages (except the row titled “All,” where the percentages are row percentages). Totals may not add up exactly to 100%.

Once an index condition is defined, the tool presents the most frequently observed comorbidities and prescriptions in separate tabs. Tables 2 and 3 present the top 5 comorbidities and prescriptions as exemplars for demonstration purposes (the default in the tool is the top 10 comorbidities and top 10 prescriptions). The individual counts are stratified by the demographic characteristics in Table 1 and shown as relative percentages. As can be seen in Table 2, “disease of the circulatory systems” is the most common comorbidity, being present in 68% of the population with gout, followed by “musculoskeletal conditions” (42%), “diseases of the genitourinary system” (39%), “diseases of the digestive system” (38%), and “diseases of the endocrine system” (36%). As can be seen in Table 3, “02 cardiovascular system” is the most common prescription by drug chapter, being present in 67% of the population with gout, followed by “10 musculoskeletal and joint disease” (45%), “01 gastrointestinal system” (36%), “04 central nervous system” (35%), and “06 endocrine system” (27%). For the top 5 comorbidities and top 5 prescriptions, people with gout over the age of 65 years have higher overall comorbidity and prescriptions than younger age groups. For example, overall, 68% of the population with gout had a comorbidity of “disease of the circulatory systems,” whereas for age groups such as “65-74” and “75-84” the proportion with the same comorbidity was much higher; 79% and 89% respectively. When compared to the overall percentages, in some cases, women showed greater than 10 percentage points difference for the comorbidity or drug chapter. For example, overall, 68% of people with gout had a comorbidity for “disease of the circulatory system,” whereas this was 79% for women. Similarly, this can be seen for “musculoskeletal conditions” (42% vs 63%), “diseases of the digestive system” (38% vs 47%), and “diseases of the endocrine system” (36% vs 51%). An exception of a lower percentage of comorbidity was observed in women with “diseases of the genitourinary system” comorbidity (39% vs 32%).

**Table 2:** An example of the top 5 comorbidities from the R Shiny application describing the distribution of patients’ characteristics, including age, gender, ethnicity, and IMD^a^ for the cohort with gout.^b^

Criteria (feature)			Top 5 comorbidities (body systems)								
			Disease of the circulatory system, n (%)		Musculoskeletal conditions, n (%)		Diseases of the genitourinary system, n (%)		Diseases of the digestive system, n (%)		Diseases of the endocrine system, n (%)
All			22,730 (68)		13,920 (42)		13,140 (39)		12,820 (38)		11,980 (36)
**Age (years)**											
	Younger than 25	0 (8)		10 (15)		0 (8)		0 (10)		0 (6)	
	25-34	50 (10)		50 (11)		50 (10)		70 (15)		60 (14)	
	35-44	380 (19)		190 (10)		220 (11)		370 (19)		340 (17)	
	45-54	1820 (37)		930 (19)		1050 (21)		1200 (25)		1150 (23)	
	55-64	4180 (60)		2260 (33)		2340 (34)		2290 (33)		2310 (33)	
	65-74	6840 (78)		4040 (46)		3960 (45)		3660 (42)		3540 (40)	
	75-84	6480 (90)		4270 (59)		3790 (53)		3590 (50)		3260 (45)	
	85 and older	2980 (94)		2180 (69)		1720 (55)		1620 (51)		1310 (41)	
**Gender**											
	Men	17,180 (65)		9500 (36)		10,920 (41)		9480 (36)		8400 (32)	
	Women	5550 (79)		4420 (63)		2220 (32)		3340 (32)		3340 (47)	
**Ethnicity**											
	Black	240 (74)		110 (34)		150 (46)		120 (37)		160 (49)	
	Chinese or Mixed or Other	240 (68)		110 (30)		150 (42)		110 (32)		120 (35)	
	South Asian	420 (68)		220 (36)		270 (44)		250 (41)		290 (47)	
	White	19,820 (73)		12,670 (47)		11,680 (43)		11,680 (43)		10,410 (38)	
	Missing	2010 (39)		810 (16)		890 (18)		660 (13)		1010 (20)	
**IMD quintile**											
	Q1 (least deprived)	5690 (65)		3280 (38)		3400 (39)		3180 (36)		2680 (31)	
	Q2	4990 (67)		3050 (41)		2930 (40)		2740 (37)		2560 (35)	
	Q3	5040 (69)		3170 (43)		2850 (39)		2780 (38)		2690 (37)	
	Q4	3930 (69)		2480 (44)		2230 (39)		2240 (39)		2240 (39)	
	Q5 (most deprived)	3070 (70)		1950 (44)		1730 (40)		1890 (43)		1800 (41)	

^a^IMD: index of multiple deprivations.

^b^For disclosure control, all numbers presented are rounded to 10, percentages are rounded to discrete numbers, and percentages presented are row percentages of the “All” column in Table 1, so totals will not be added exactly.

**Table 3:** An example of the top 5 prescriptions from the R Shiny application describing the distribution of patients’ characteristics, including age, gender, ethnicity, and IMD^a^ for the cohort with gout.^b^

Criteria (feature)			Top 5 prescriptions (drug chapters)								
			02 cardiovascular system, n (%)		10 musculoskeletal and joint disease, n (%)		01 gastrointestinal system, n (%)		04 central nervous system, n (%)		06 endocrine system, n (%)
All			22,310 (67)		14,920 (45)		11,980 (36)		11,720 (35)		8980 (27)
**Age (years)**											
	Younger than 25	0 (4)		10 (19)		0 (8)		10 (19)		0 (4)	
	25-34	40 (9)		120 (26)		50 (10)		70 (14)		30 (6)	
	35-44	330 (17)		630 (32)		260 (13)		380 (19)		160 (8)	
	45-54	1660 (34)		1880 (38)		1000 (20)		990 (20)		660 (13)	
	55-64	4080 (59)		3070 (44)		2010 (29)		2000 (29)		1490 (22)	
	65-74	6960 (79)		4320 (49)		3370 (38)		3110 (35)		2590 (29)	
	75-84	6390 (89)		3510 (49)		3540 (49)		3430 (48)		2760 (38)	
	85 and older	2840 (90)		1380 (44)		1740 (55)		1740 (55)		1300 (41)	
**Gender**											
	Men	16,900 (64)		12,030 (45)		8690 (33)		7820 (30)		5960 (23)	
	Women	5410 (77)		2900 (41)		3290 (47)		3890 (55)		3020 (43)	
**Ethnicity**											
	Black	220 (70)		140 (43)		110 (33)		110 (34)		110 (34)	
	Chinese or Mixed or Other	240 (66)		170 (48)		100 (29)		100 (28)		100 (28)	
	South Asian	400 (65)		270 (44)		250 (40)		240 (39)		230 (38)	
	White	19,370 (71)		12,550 (46)		10,930 (40)		10,660 (39)		7940 (29)	
	Missing	2080 (41)		1790 (35)		590 (12)		620 (12)		600 (12)	
**IMD quintile**											
	Q1 (least deprived)	5560 (64)		3660 (42)		2710 (31)		2390 (27)		2000 (23)	
	Q2	4910 (66)		3270 (44)		2500 (34)		2440 (33)		1980 (27)	
	Q3	4950 (68)		3260 (45)		2740 (38)		2550 (35)		2060 (28)	
	Q4	3900 (69)		2670 (47)		2230 (39)		2280 (40)		1620 (29)	
	Q5 (most deprived)	2990 (68)		2070 (47)		1800 (41)		2060 (47)		1320 (30)	

^a^IMD: index of multiple deprivations.

^b^For disclosure control, all numbers presented are rounded to 10, percentages are rounded to discrete numbers, and percentages presented are row percentages of the “All” column in Table 1, so totals will not be added exactly.

Different ethnic groups with gout showed different percentages of comorbidities for different diseases. For example, 47% of the White ethnic group had a musculoskeletal condition comorbidity, whereas this was only 34% of those within the Black ethnic group. However, the comorbidity of “diseases of the endocrine system” was observed in 49% of the Black ethnic group in comparison to 38% of the White ethnic group. There was a general observation that the higher the level of deprivation, the higher the percentage of the population with each comorbidity and prescription.

Such differences are easily highlighted in the tool for guideline developers aiding their workflows regardless of index conditions and parameters specified.

Alternatively, it is possible to view the most frequent comorbidities and prescriptions at the lower level of granularity given in the hierarchical groupings (ie, comorbidities as condition groups and/or individual conditions and prescriptions as BNF classes and/or individual drugs).

In addition to viewing the most common top-level comorbidities and prescriptions for the condition of interest (gout in this case), there is an option for the user to select comorbidities and prescriptions of interest from the complete list of comorbidities and prescriptions. This user selection feature is supported at each hierarchical level of comorbidities (ie, body system, condition group, and individual condition) and prescriptions (ie, drug chapter, drug class, and individual drug). Table 4 demonstrates user selection of comorbidities at the “condition group” level and prescriptions at “drug class” levels. Selected drug class level statistics were requested by the NICE gout GDG. There were similar trends observed in the data for this lower level of granularity.

**Table 4:** An example of the user-selected comorbidities (ie, condition groups) and prescriptions (drug classes) from the R Shiny application describing the distribution of patients’ characteristics, including age, gender, ethnicity, IMD^a^, and time since diagnosis (prevalent and recently incident populations).^b^

Criteria (feature)		User-selected comorbidities (condition groups)					User-selected prescriptions (drug classes)			
		Hypertension, n (%)	Ulcer and upper gastrointestinal acid conditions, n (%)	Chronic lung disease, n (%)	Coronary heart disease, n (%)	Statins, n (%)		Angiotensin receptor blockers, n (%)	Oral anticoagulants, n (%)	Thiazide diuretics, n (%)
All		20,540 (61)	12,120 (36)	7260 (22)	6980 (21)	13,740 (41)		4750 (14)	3380 (10)	3050 (9)
**Age (years)**										
	Younger than 25	0 (4)	0 (8)	20 (35)	0 (0)	0 (0)		0 (0)	0 (0)	0 (0)
	25-34	30 (6)	60 (13)	110 (24)	0 (1)	10 (3)		0 (0)	0 (1)	0 (0)
	35-44	300 (16)	340 (17)	360 (18)	30 (2)	120 (6)		40 (2)	10 (1)	20 (1)
	45-54	1590 (32)	1090 (22)	840 (17)	240 (5)	820 (17)		280 (6)	90 (2)	150 (3)
	55-64	3710 (54)	2100 (30)	1270 (18)	800 (12)	2430 (35)		790 (11)	270 (4)	520 (8)
	65-74	6210 (71)	3470 (39)	1970 (22)	2050 (23)	4740 (54)		1560 (18)	920 (10)	990 (11)
	75-84	5940 (82)	3470 (48)	1880 (26)	2560 (35)	4120 (57)		1510 (21)	1420 (20)	1000 (14)
	85 and older	2760 (87)	1580 (50)	830 (26)	1300 (41)	1500 (48)		570 (18)	660 (21)	360 (11)
**Gender**										
	Men	15,360 (58)	8920 (34)	5230 (20)	5470 (21)	10,812 (41)		3369 (13)	2593 (10)	2059 (8)
	Women	5190 (74)	3190 (45)	2030 (29)	1520 (22)	2931 (42)		1380 (20)	791 (11)	989 (14)
**Ethnicity**										
	Black	230 (72)	100 (33)	70 (21)	60 (17)	130 (39)		50 (17)	20 (7)	50 (15)
	Chinese or Mixed or Other	210 (58)	100 (29)	60 (16)	70 (20)	160 (43)		50 (14)	20 (6)	30 (8)
	South Asian	380 (62)	230 (37)	150 (24)	160 (27)	280 (46)		110 (18)	30 (4)	50 (9)
	White	17,850 (66)	11,080 (41)	6380 (24)	6560 (24)	12,000 (44)		4140 (15)	3250 (12)	2610 (10)
	Missing	1880 (37)	600 (12)	610 (12)	140 (3)	1180 (23)		400 (8)	60 (1)	310 (6)
**IMD quintile**										
	Q1 (least deprived)	5100 (58)	2990 (34)	1630 (19)	1630 (19)	3320 (38)		1300 (15)	870 (10)	780 (9)
	Q2	4500 (61)	2590 (35)	1510 (20)	1470 (20)	2990 (40)		1130 (15)	770 (10)	640 (9)
	Q3	4560 (62)	2640 (36)	1570 (22)	1600 (22)	3070 (42)		1030 (14)	750 (10)	700 (10)
	Q4	3590 (63)	2110 (37)	1370 (24)	1240 (22)	2380 (42)		760 (13)	570 (10)	530 (9)
	Q5 (most deprived)	2800 (64)	1790 (41)	1180 (27)	1040 (24)	1980 (45)		520 (12)	420 (10)	400 (9)

^a^IMD: index of multiple deprivations.

^b^For disclosure control, all numbers presented are rounded to 10, percentages are rounded to discrete numbers, and percentages presented are row percentages of the “All” column in Table 1, so totals will not be added exactly.

**Figure 4:**
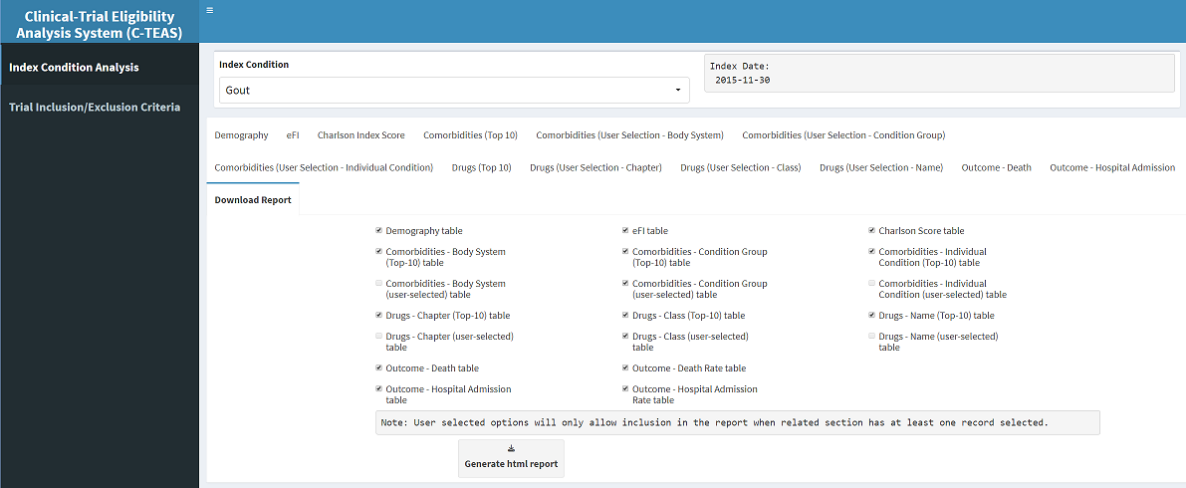
A screenshot of the report generation page.

As per the requirements, the tool supports the generation of printable reports in HTML format. The individual outcomes to save and print (and then save as PDF) are user selectable, as shown in Figure 4.

### Clinical Trial Population Analysis

**Figure 5:**
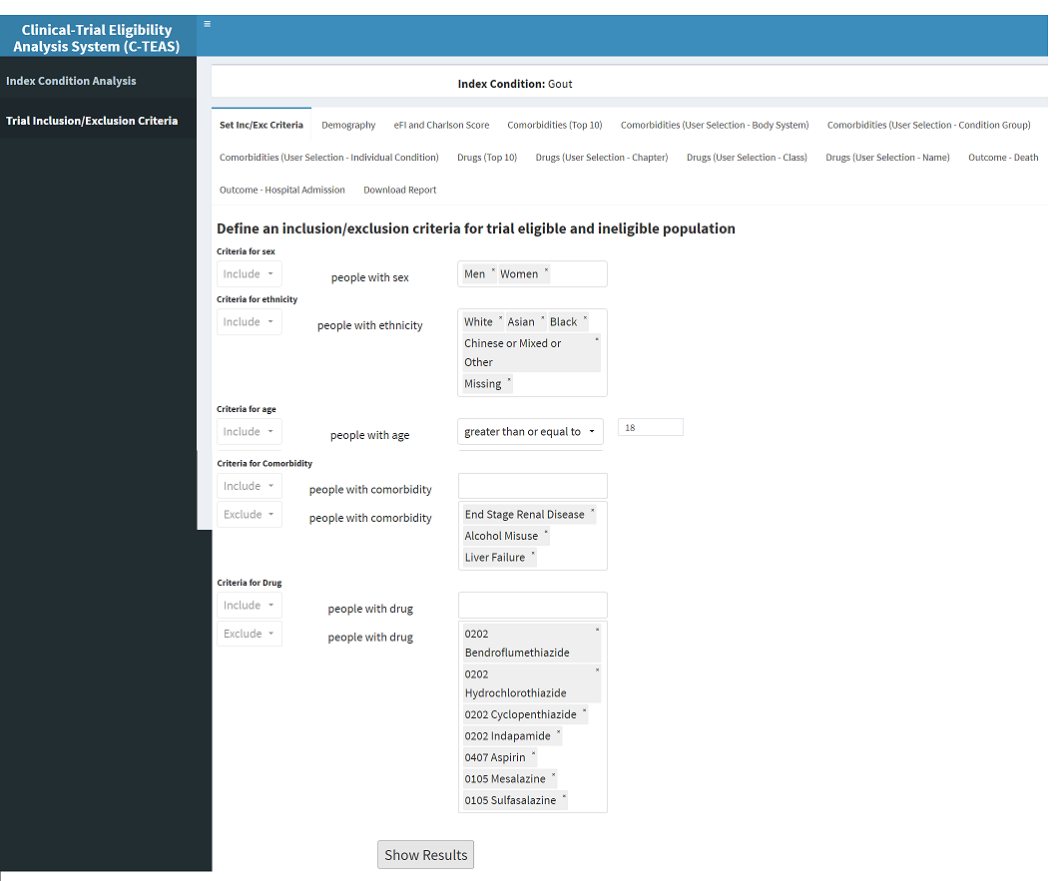
A screenshot of the R Shiny tool. Setting up inclusion and/or exclusion criteria to analyze eligible and ineligible populations.

The R Shiny tool enables comparisons between trial eligible and ineligible populations using inclusion and exclusion criteria. As illustrated in Figure 5, a user can set inclusion and exclusion criteria based on five main criteria: gender, ethnicity, age, comorbidities (only for individual conditions from hierarchical groupings of conditions), and drug prescription (only for individual drugs).

The results of a trial eligible and ineligible query are shown in Table 5. The criteria were adapted as best possible given the different sources of data from a published gout trial in the literature [38].

**Table 5:** Output generated for an example trial taken from the literature.^a^

Criteria (feature)			All, n (%)		Eligible, n (%)		Ineligible, n (%)
All			33,480		27,080 (81)		6400 (19)
**Age (years)**							
	Younger than 25	50 (0)		40 (85)		10 (15)	
	25-34	470 (1)		420 (91)		40 (9)	
	35-44	1960 (6)		1740 (89)		220 (11)	
	45-54	4900 (15)		4220 (86)		680 (14)	
	55-64	6930 (21)		5580 (81)		1350 (19)	
	65-74	8800 (26)		6850 (78)		1960 (22)	
	75-84	7210 (22)		5610 (78)		1600 (22)	
	85 and older	3160 (9)		2610 (83)		550 (17)	
**Gender**							
	Men	26,440 (79)		21,520 (81)		4920 (19)	
	Women	7040 (21)		5560 (79)		1480 (21)	
**eFI^b^**							
	Fit	13,650 (41)		12,020 (44)		1630 (25)	
	Mild frailty	10,220 (31)		7850 (29)		2370 (37)	
	Moderate frailty	6210 (19)		4620 (17)		1590 (25)	
	Severe frailty	3400 (10)		2600 (10)		810 (13)	
**Comorbidities (condition group)—user selected**							
	Hypertension	20,540 (61)		15,320 (57)		5230 (82)	
	Ulcer and upper gastrointestinal acid conditions	12,120 (36)		9330 (34)		2780 (43)	
	Chronic lung disease	7260 (22)		5600 (21)		1660 (26)	
	Coronary heart disease	6980 (21)		5370 (20)		1620 (25)	
**Prescriptions (drug class)—user selected**							
	Statins	13,740 (41)		10,400 (38)		3340 (52)	
	Angiotensin receptor blockers	4750 (14)		3450 (13)		1300 (20)	
	Oral anticoagulants	3380 (10)		2660 (10)		720 (11)	
	Thiazide diuretics	3050 (9)		70 (0)		2980 (47)	

^a^For disclosure control, all numbers are rounded to 10, percentages are rounded to discrete numbers, and the percentages are column-wise percentages except for a special case of age and gender where percentages in the eligible and ineligible columns are row-wise percentages based on the totals (for each age group or gender type) in the column “All”), and so totals will not add exactly.

^b^eFI: electronic frailty index.

The inclusion criteria are as follows: (1) age (18 years and older); (2) gender (men and women); and ethnicity (any). The exclusion criteria are as follows: (1) comorbidities (end-stage renal disease, alcohol misuse, and liver failure) and (2) drugs (bendroflumethiazide, hydrochlorothiazide, cyclopenthiazide, indapamide, aspirin, mesalazine, and sulfasalazine).

Table 5 illustrates the output collated from multiple output tabs of the tool, capturing demographics, eFI, comorbidities (user selected), and prescriptions (user selected) in terms of trial eligible and ineligible populations. Overall, 81% of the population with gout were eligible, and 19% were ineligible based upon the specified inclusion and exclusion criteria—the current version of the tool is a proof-of-concept and supports a subset of the criteria specified in the published gout trial [38]. In comparing the eligible and ineligible columns, the trialist can assess biases between the cohorts regarding any inherent demographic, comorbidity (commonest and user selected), and prescriptions (commonest and user selected). It should be noted that the total percentage within the eFI, comorbidities, and prescriptions in eligible and ineligible columns do not add up to 100% (as they do for “All,” “Age (years),” and “Gender” groupings) as individuals may have less than 1 or 0 comorbidities or prescriptions, and the results presented are not as a proportion of all of the population with gout (as they are for “All,” “Age (years),” “Gender,” and “eFI” groupings) against the “All” column instead they are based on total eligible and ineligible populations.

### Integrated Disclosure Control and Report Generation

Accidental disclosure of low-count data was avoided by built-in rules, which rounded values to the nearest 10 and percentages to integers. The integrated disclosure-control mechanism was not only helpful in ensuring patients’ confidentiality when guideline users were conducting analysis using the tool within the TRE but also enabled rapid egressing of the reports from the TRE—as the TRE output checking staff already knew that outputs generated from the tool were nondisclosive.

Completed analyses can be saved as reports. Printable versions of the completed results across tabs for the overall population and trial eligible or ineligible population analyses are presented in Multimedia Appendices 4 and 5, respectively.

### Guideline Users Feedback

To evaluate the tool’s functionality, several users from NICE and SIGN guideline developers were given access to the tool within HIC TRE. They found the tool to be very useful while developing guidelines to enable the use of real-world evidence about the nature of the population for whom different treatments were being recommended. They have shown significant interest in the R Shiny tool in its current form due to its simplest navigation functionality for generating analysis and reports at pace and in further development into a production version. The tool supports many different outputs, for example, demography statistics, top comorbidities statistics, user-selected comorbidities, and prescription statistics. There was a general consensus across users testing the functionality that the generation of a range of outputs for the chosen disease/condition within a few minutes is highly advantageous. One of the users (ie, a health economist from NICE) was of the view that generating statistics on comorbidities could be helpful for the study of the burden of comorbidities on the health systems. One of the users of the tool from NICE expressed his views about the tool:

The tool enables us to assess the relevance of RCTs and observational studies to real-world UK patients, by comparison between real-world patient characteristics and study eligibility criteria. This provides a new lens through which committees and developers could critically assess studies captured in evidence reviews. There are interesting potential applications of the tool inform surveillance, scoping, and NICE research recommendations. The tool is interactive and returns results immediately. This maximises its potential impact for guideline development, as extended waits for data access and results may mean analyses come too late to be usefully considered by guideline committees working to tight deadlines. The clear format, where most common conditions and prescriptions can be viewed at a level of granularity chosen by the user, is ideal. NICE’s 2021-26 Strategy emphasizes increased use of real-world evidence and more rapid and responsive guideline development. This tool aligns well with these ambitions. We hope there will be opportunities to further test the tool in guideline development in future.

User #1, male

### Key Features of the Tool

Factors such as ease of use of the tool by novice users, the query processing time to extract the required data, integral disclosure control mechanism, and standardized outputs across various chronic conditions were among the challenges addressed when developing the application. The tool is simple to use, with only 2 menu options and minimum input required to generate results for both types of analysis using a graphical interface. To optimize the performance of query processing, we designed an intermediate data structure populated by the data from the CPRD GOLD subset. This resulted in retrieving results from each type of analysis within approximately a couple of minutes compared to a much longer time when running a query against the raw CPRD GOLD data set (eg, generating population-level results at the demography level took more than 3-5 minutes running on the raw data set versus only a few seconds on the optimized data structure). The outputs were designed in consultation with the project advisory group and the gout GDG, but they are generalizable to other chronic conditions and are usable by other GDGs.

### Limitations of the Tool

The tool has limitations in its current form: (1) it only allows index condition selection at the lower level in the hierarchical groupings of conditions (such as gout, asthma, and atrial fibrillation); (2) trial criteria selection only allows age, gender, ethnicity, comorbidities (lower level in the hierarchical groupings of conditions), and prescriptions (individual drug names, for instance, bendroflumethiazide, aspirin, and mesalazine); (3) only statistics around hospitalization and mortality are included (and not the patients outcomes from hospitalization and mortality); (4) there are limits to demography-based cross-tabulation across all output for a selected index condition; (5) the tool only includes primary care data for generating evidence; (6) the data it uses are retrospective and relies on Read codes which are being retired in UK primary care electronic records, but with mapping to Systematized Medical Nomenclature for Medicine–Clinical Terminology which is replacing them; (7) it does not allow users to extract information based on user-defined disease definitions (phenotype algorithms); instead it uses predefined disease definitions (phenotype algorithms); (8) users are required to access the tool within a TRE for generating evidence although it generates aggregate level information which meets disclosure control mechanisms in place; and (9) it performs slower for the diseases or conditions that have bigger cohort sizes, even with a subset of around 1 million patient data using an optimized data structure.

## Discussion

### Principal Findings

This study describes a proof-of-concept tool for analyzing clinical populations with various comorbidities in terms of the overall population and trial analysis. The tool was developed for use by guideline developers to develop clinical evidence–driven guidelines, using the specific example of gout. Typically, clinical guideline recommendations are developed using the evidence available in the published literature, mainly relying on RCT results or, in case of nonexistence of such studies, the experience of the people developing guidelines is used when preparing guideline recommendations [39].

The WHO has envisaged a future that uses clinical data in the development of recommendations [40]. The WHO’s Standard-based, Machine-readable, Adaptive, Requirements-based, and Testable framework focuses on transformation from paper-based systems to smart digital systems. Recently, the focus has been shifted to computer-interpretable guideline development, highlighting the need for EHR integration with clinical practice guidelines as one of the emerging themes [41]. However, the challenges of using the computer-interpretable guidelines for patients with multimorbidity would be complex [42].

The tool described here is intended to be used by guideline developers with minimal understanding of the structure or coding of the underlying data, which makes it distinct from tools like Atlas, which are more flexible because users can define cohorts in detail, but require greater baseline knowledge and skills [43]. In the longer term, large guideline developers like NICE might find the flexibility of tools like Atlas helpful, but it is likely that smaller guideline developers with fewer resources would benefit from having access to tools that require less baseline knowledge and skills. The tool developed here addresses the issue of generating evidence from a relevant clinical population by using linked EHR data, thereby assisting guideline developers in better understanding the clinical population and making guideline recommendations. The use of such a tool can enhance the quality of the guidelines, which have often struggled to be generalizable in terms of external validity or lack of evidence from the clinical population.

Future development will focus on: (1) supporting the selection of the index condition at a higher level in the hierarchical groupings (ie, condition group and body system); (2) enhancement of the choice of trial eligibility criteria (eg, laboratory tests), more complex combination of comorbidities and prescription selection such as allowing user to select comorbidities (ie, body system, condition group, and individual condition) and prescriptions (ie, drug chapter, drug class and individual drug) with different hierarchal levels both for inclusion and exclusion criteria; (3) inclusion of outcomes data of hospitalization and mortality; (4) allowing user choice of cross-tabulation as the current form of the tool cross-tabulate everything by demography automatically (for example, data on prescribing drugs for gout be stratified by chronic kidney disease stages 3-5 and non-chronic kidney disease population stage 1-2); (5) for some guidelines, primary care is not the ideal source of data, so enhancement of the tool to query other data sets (eg, rare disease registries or specialist routine care data sets); (6) capability of supporting different coding systems other than Read code/ICD10 for disease or condition definitions using the ontological approach, such as Read codes mapping to Systematized Medical Nomenclature for Medicine-Clinical Terminology, and prescriptions based on BNF (or matching international standards to BNF); (7) enabling the capability of bringing disease or condition cohort definition algorithms other than the listed definitions supported by the tool; (8) enabling the tool to save outputs using and load results offline for analyzing within and outside the TRE after the required governance mechanism in place results from the precomputed instead of using individual patient-level data; (9) making the codebase of the tool publicly available after cleaning it, so researchers interested in generating evidence from the EHR data could benefit. As of now, the codebase in its current form is available on request; and (10) the majority of RCTs in people with physical conditions exclude more than half of the patients with the target condition. The tool is developed as a proof-of-concept to facilitate guideline developers generating evidence from the routinely collected primary care clinical data [8]. This proof-of-concept tool was intended to support guideline developers to better understand how to generalize RCT findings to the wider population, but future work could also explore its use by trialists to understand the implications of the choice of inclusion or exclusion criteria for generalizability.

We have provided access to the tool for assessment by the guideline users from NICE and SIGN. Their qualitative feedback was very encouraging, and they viewed the tool as very useful, but further evaluation of use is needed. They like to use it as an integral part of the guideline development process. The tool was developed as a proof of concept rather than a production tool. We are currently assessing how we can develop the tool further so that it can be provided as a service for guideline development groups across the United Kingdom to make more effective guideline recommendations based on evidence generated from the clinical population.

### Conclusions

We have demonstrated a tool for generating evidence from clinical populations that could be used to inform clinical guideline development. The challenge of query performance on the raw CPRD GOLD data source was resolved by designing and implementing an optimized database structure. An exemplary case study of gout demonstrated that large sets of data relevant to and tailored to guideline development can be rapidly generated using the tool. The requirements for the prototype tool were determined with input from guideline developers. The final prototype version was evaluated by the guideline developers from NICE and SIGN. They responded positively and discussed how this could be made available as a service for them, which was not under the project’s remit due to governance approvals in place for the study. Despite the mentioned limitations, the tool is already capable of reducing data bottlenecks of guidance development.

## Appendix

Multimedia Appendix 1 Supplementary material S1.

Multimedia Appendix 2 Supplementary material S2.

Multimedia Appendix 3 Supplementary material S3.

Multimedia Appendix 4 Supplementary material S4.

Multimedia Appendix 5 Supplementary material S5.

## Abbreviations

BNF: British National Formulary
CPRD: Clinical Practice Research Datalink
eFI: electronic frailty index
EHR: electronic health record
GDG: guideline development group
GRADE: Grades of Recommendation Assessment, Development and Evaluation
HIC: Health Informatics Centre
ICD10: International Classification of Diseases 10th Revision
IMD: index of multiple deprivations
NICE: National Institute for Health and Care Excellence
RCT: randomized controlled trial
SIGN: Scottish Intercollegiate Guidelines Network
TRE: Trusted Research Environment
WHO: World Health Organization
