# Supplementary material for: A Digital Tool for Clinical Evidence–Driven Guideline Development by Studying Properties of Trial Eligible and Ineligible Populations: Development and Usability Study
Source: J Med Internet Res. 2025 Jan 16;27:e52385. doi: 10.2196/52385 (PMC11783027; doi:10.2196/52385)
Supplement: Multimedia Appendix 1 [file jmir_v27i1e52385_app1.pdf]

Table Name: Tool_Patients_demography			
**Sr. No.**	**Field Name**	**Data Type**	**Description**
1	patid	int	Encrypted unique identifier given to a patient in CPRD GOLD
2	gender	int	Gender (1=men and 2=women)
3	yob	int	Year of birth
4	tod	date	Transfer out date (left GP practice)
5	age_at_index	float	Age at index (2015 - yob)
6	AgeGroupI	varchar(255)	Age group at index
7	deathdatePC	date	Death date according to primary care
8	deathdateONS	date	Death date according to ONS
9	finalEthnicity	varchar(255)	According to lookup (defined according to CALIBER - take most recently recorded Primary Care readcode). If not recorded in Primary Care, use the ethnicity recorded in HES. The field has distinct values: Bangladeshi, Black Afican, Black Caribbean, Black Other, Chinese, Indian, Mixed, Other, Other Asian, Pakistani, Unknown and White
10	finalEthnicity5cat	varchar(255)	Grouped ethnicity into 5 categories (i.e. Asian or Asian British; Black, African, Caribbean or Black British; Chinese, Mixed or other ethnic group; Unknown ethnic group; White)
11	finalEthnicity6cat	varchar(255)	Grouped ethnicity into 6 categories (i.e. Asian or Asian British; Black, African, Caribbean or Black British; Chinese or other ethnic group; Mixed or Multiple ethnic groups, Unknown ethnic group)
12	IMD_quintile	varchar(255)	IMD at 2015, divided into fifths. (Patient residence, not practice). Patients without deprivation have been removed from the cohort.
13	IMD_decile	varchar(255)	IMD at 2015, divided into ten. (Patient residence, not practice). Patients without deprivation have been removed from the cohort.
14	HospitalFrailtyIndex	float	scored
15	HospitalFrailtyCategory	varchar(255)	category of score
16	CharlsonScore	int	Charlson Score
17	eFICategory	varchar(255)	eFI Category
18	status	float	Status = 1 (censored at 30/11/2018 or transfer out), Status = 2 (died during follow up)
19	FUend	date	End date for follow up
20	FUtime	float	number of years follow-up
21	n_DNA_records	float	number of DNA (did not attend) in the year prior to index. Excludes did not attends for vaccinations.
22	Care_Residential_home	float	1 = believe to live in the care home
23	CharlsonScoreCat	varchar(255)	Category of Charlson Score 2019

Table Name: Tool_Clinical			
Sr. No.	Fields	Data Type	Description
1	patid	int	Encrypted unique identifier given to a patient in CPRD GOLD
2	CategoryLevel3BG	varchar(255)	Disease categories at body system level
3	IntermediateLevel2	varchar(255)	Disease categories at condition group level
4	ConditionLevel1	varchar(255)	Disease categories at individual condition level
5	HistoryFlag	float	History flag = 1 (medcode suggests that this is a historical diagnosis, recorded on the first record in primary (i.e. this patient has previously been diagnosed with X, usually indicative of being diagnosed in a different practice, so we don't know the original diagnosis date)
6	date_of_pc_diagnosis	date	date first recorded in primary care
7	ComorbidityList	varchar(255)	Use as an comorbidity of interest in the tool
8	IndexList	varchar(255)	Use as an index condition in the list
9	date_of_first_admission	date	Date of first hospital admission (any ICD)
10	date_of_last_admission	date	Date of last hospital admission (any ICD)
11	HospitalisedInPrev5y	float	Hospitialisation in Previous 5 Years = 1 (if data of admission >=01/12/2010)
12	diagnosis_date	date	Minimum date of pc_diagnosis and date of first admissions
13	incident_cohort	float	incident_cohort=1 (this diagnosis was first recorded in the year prior to the index (01/12/2014 - 30/11/2015), and was not a "History of X" code (common in medcodes))
14	PrimaryHosp1yr	float	Had a hospital for this cause in the Primary ICD position in the year prior to index date
15	date_of_last_Primary_admission	date	Date of hospitalisation for this cause cause in the primary ICD position

Table Name: Tool_Therapy			
Sr. No.	Fields	Data Type	Description
1	patid	int	Encrypted unique identifier given to a patient in CPRD GOLD
2	most_recent_prescription	date	most recent date for the prescription
3	Name	varchar(255)	Drug name
4	Class	varchar(255)	BNF classification
5	ClassFullCode	varchar(255)	BNF classification code
6	specialFlag	float	a special drug (like aspirins)
7	CombinationDrug	float	Drugs with different name, that are a combination (so share the same prodcode)
8	formulation_group	varchar(18)	Oral, eye/nose.ear etc. Mainly used to identify oral versions of drugs for exclusions
9	avg_current_dose	float	only for prednisone/prednisilone as there are exclusions for high dosages
10	Name_current_prescr	int	Name_current_prescr = 1 (Prescribed in the 84 days prior to index (or 168 days for Warfarin or insulin) issued 07/09/2015 - 30/11/2015)
11	Name_chronic_prescr	int	Name_chronic_presc = 1 (Prescribed in the 84/168 days prior to a current prescr and has current prescription)
12	Name_total_prescriptions	int	Total number of prescription (for drug name)
13	Class_current_prescr	int	Class_current_prescr = 1 (Prescribed in the 84 days prior to index (or 168 days for Warfarin or insulin) issued 07/09/2015 - 30/11/2015)

14	Class_chronic_prescription	int	Class_chronic_presc = 1 (Prescribed in the 84/168 days prior to a current prescr and has current prescription)
15	Class_total_prescriptions	int	Total number of prescription (for drug class)
16	Chapter	varchar(53)	BNF chapter
17	NameClass	varchar(255)	Combination of drug name and the first four digits of their drug class code to allow for differentiation between Aspirin from multiple chapter etc.

Table Name: Tool_Outcome_death			
Sr. No.	Fields	Data Type	Description
1	patid	int	Encrypted unique identifier given to a patient in CPRD GOLD
2	FUtime	float	Amount of time followed up in years (from 30/11/2015 to maximum of 30/11/2018), their death or their transfer out date)
3	FUend	date	End date for follow up
4	status	float	Status =1 (censored at end of follow-up/transfer out), Status = 2 (death)
5	cause	varchar(255)	ICD code (main)
6	ICD_3dig	varchar(255)	Three digits of ICD code
7	major	varchar(255)	Major ICD
8	sub_chapter	varchar(255)	Subchapter of ICD
9	chapter	varchar(255)	ICD chapter
10	BG_AND_DM_comment	varchar(255)	Flags if the ICD is worth including in the tree or if only the chapter and subchapter should be included in the major
11	High_level_category	varchar(255)	Special groupings defined by expertes BG and DM
12	Core_condition_group	varchar(255)	Special groupings defined by expertes BG and DM
13	Name	varchar(255)	Condition Name
14	BG	varchar(255)	Comments from BG

Table Name: Tool_Outcome_hosp			
Sr. No.	Fields	Data Type	Description
1	patid	int	Encrypted unique identifier given to a patient in CPRD GOLD
2	FUend	varchar(255)	End date for overall follow-up
3	Futime	float	Amount of time followed up in years (from 30/11/2015 to maximum of 30/11/2018, their death or transfer out date)
4	status	float	Status = 1 (Censored at end of follow up/Transfer out date), Status = 2 (died)
5	major	varchar(255)	ICD major cause
6	maj_date	date	First admission date for the ICD major cause
7	n_major_admit	int	Number of admissions during follow-up for that major cause
8	sub_chapter	varchar(255)	ICD subchapter cause (grouped majors)
9	sub_chpt_date	date	Minimum date of relevant ICD majors, grouped into subchapter
10	n_subchpt_admit	int	Number of admissions during follow-up for that ICD subchapter
11	chapter	varchar(255)	ICD chapter cause (grouped subchapters)
12	chpt_date	date	Minimum date of relevant ICD subchapters
13	n_chpt_admit	int	Number of admissions during follow-up for that ICD chapter
14	BG_AND_DM_comment	varchar(255)	Comments from BG and DM
15	FUtime_major	float	FUtime for ICD major
16	FUtime_sub	float	FUtime for ICD sub
17	Futime_chpt	float	FUtime for ICD chapter
