# Supplementary material for: A Digital Tool for Clinical Evidence–Driven Guideline Development by Studying Properties of Trial Eligible and Ineligible Populations: Development and Usability Study
Source: J Med Internet Res. 2025 Jan 16;27:e52385. doi: 10.2196/52385 (PMC11783027; doi:10.2196/52385)
Supplement: Multimedia Appendix 2 [file jmir_v27i1e52385_app2.pdf]

**Supplementary Material S2**

*Table S2-1: An overview of analysis types and specifying inputs and outputs for the R shiny application*

| Analysis Type | Input | Output<br>(All the outputs are presented in terms of numbers and percentages) | Comments |
|---|---|---|---|
| Population Analysis | Selection of chronic condition from the given list | Demography features analysis<br>• Age distribution<br>• Sex distribution<br>• Ethnicity distribution<br>• Indices of multiple deprivation (IMD)<br>• Prevalent and incident distributions | |
| | | Electronic Frailty and Charlson Comorbidity Score analysis<br>• electronic Frailty Index (eFI) score distributions based on demography features such as overall, age, sex, ethnicity and IMD<br>• Charlson Comorbidity score distributions based on demography features such as overall, age, sex, ethnicity and IMD | |
| | | Comorbidity Analysis<br>• Top-10 comorbidities – in terms of features such as overall, age, sex, ethnicity and IMD<br>• User selected comorbidities – in terms of features such as overall, age, sex, ethnicity and IMD | The tool supports comorbidities analysis at three hierarchal levels based on user selection (i.e., Body System, group conditions and individual conditions). For example, Cancers is a body system; and it has three condition groups such as haematological cancers, solid organ cancer – primary and solid organ cancer – secondary; and similarly condition group of haematological cancer has individual conditions such as Hodgkins Lymphoma, Leukomia, Myelodysplastic syndrome, non hodgkins lymphoma, plasma cell malignancy and polycythaemia vera. |
| | | Co-prescription Analysis<br>• Top-10 co-prescription – in terms of features such as overall, age, sex, ethnicity and IMD<br>• User selected co-prescription – in terms of features such as overall, age, sex, ethnicity and IMD | The tool supports co-prescriptions analysis at three hierarchy levels based on user selection such as drug chapter, drug class and drug name. For example, drug chapter is ’02 Cardiovascular System’, drug classes are ‘ACE Inhibitors’, ‘Omega-3 fatty acids’ and drug name are ‘0205 Captopril’, ‘0205 Enalapril’. |
| | | Hospitalisation Analysis | |

| | | | |
|---|---|---|---|
| | | • Hospital admissions in terms of one year, two year and three years follow up time from the index date (i.e., 30.11.2015)<br>• Hospital admission rate distributions | |
| | | Mortality Analysis<br>• Deaths in terms of one year, two year and three years follow up time from the index date (i.e., 30.11.2015)<br>• Death rate distributions | |
| | | Dynamic report generation | A user has an option to select which analysis results needs to be printed as an HTML report. In terms of wider tables, the tool automatically breaks it down to multiple tables. |
| Clinical Trial Analysis | Selection of trial criteria:<br>• Gender<br>• Ethnicity<br>• Age<br>• Comorbidity (inclusion and exclusion both)<br>• Drug (inclusion and exclusion). | An output to this component is similar to population analysis output but gives output in terms of trial eligible and ineligible populations. This includes demography analysis, eFI and charlson score analysis, comorbidity analysis, co-prescription analysis, hospitalisation and mortality analysis and dynamic report generation. | • For gender and ethnicity, the tool allows to keep the values to be included and delete the values that needs to be excluded.<br>• For age, the tool allows the use of relational operators such as greater than, greater than or equal to, less than, less than or equal to, not equal to, equal to and between (with range starting and ending values inclusive or not)<br>• Dynamic report generation allowing users to select which tables to include in the report. |
