# Supplementary material for: A Digital Tool for Clinical Evidence–Driven Guideline Development by Studying Properties of Trial Eligible and Ineligible Populations: Development and Usability Study
Source: J Med Internet Res. 2025 Jan 16;27:e52385. doi: 10.2196/52385 (PMC11783027; doi:10.2196/52385)
Supplement: Multimedia Appendix 3 [file jmir_v27i1e52385_app3.pdf]

Supplementary Material S3

Conditions Hirarchy - R Shiny Tool<br>(List of conditions to be supported as index condition (N=128) and comorbidities (N=161) in the R shiny tool)<br>Note: The current version of the r shiny application allows use of individual conditions to be selected as index condition whereas comorbidities can be selected either as body system or as condition group or as individual condition					
**Sr No**	**Body System**	**Condition Group**	**Individual Condition**	**Inclusion as Index Condition**	**Inclusion as Comorbidity**
1	Cancers	Haematological cancer	Hodgkins Lymphoma	Y	Y
2	Cancers	Haematological cancer	Leukaemia	Y	Y
3	Cancers	Haematological cancer	Myelodysplastic Syndrome	Y	Y
4	Cancers	Haematological cancer	Non Hodgkins Lymphoma	Y	Y
5	Cancers	Haematological cancer	Plasma Cell Malignancy	Y	Y
6	Cancers	Haematological cancer	Polycythaemia vera	Y	Y
7	Cancers	Solid organ cancer - primary	Primary Malignancy - Biliary	Y	Y
8	Cancers	Solid organ cancer - primary	Primary Malignancy - Bladder	Y	Y
9	Cancers	Solid organ cancer - primary	Primary Malignancy - Bone	Y	Y
10	Cancers	Solid organ cancer - primary	Primary Malignancy - Bowel	Y	Y
11	Cancers	Solid organ cancer - primary	Primary Malignancy - Brain	Y	Y
12	Cancers	Solid organ cancer - primary	Primary Malignancy - Breast	Y	Y
13	Cancers	Solid organ cancer - primary	Primary Malignancy - Cervix	Y	Y
14	Cancers	Solid organ cancer - primary	Primary Malignancy - Kidney	Y	Y
15	Cancers	Solid organ cancer - primary	Primary Malignancy - Liver	Y	Y
16	Cancers	Solid organ cancer - primary	Primary Malignancy - Lung	Y	Y
17	Cancers	Solid organ cancer - primary	Primary Malignancy - Melanoma	Y	Y
18	Cancers	Solid organ cancer - primary	Primary Malignancy - Mesothelioma	Y	Y
19	Cancers	Solid organ cancer - primary	Primary Malignancy - Multiple Sites	Y	Y
20	Cancers	Solid organ cancer - primary	Primary Malignancy - Oesophageal	Y	Y
21	Cancers	Solid organ cancer - primary	Primary Malignancy - Oropharyngeal	Y	Y
22	Cancers	Solid organ cancer - primary	Primary Malignancy - other	Y	Y
23	Cancers	Solid organ cancer - primary	Primary Malignancy - Ovary	Y	Y
24	Cancers	Solid organ cancer - primary	Primary Malignancy - Pancreas	Y	Y
25	Cancers	Solid organ cancer - primary	Primary Malignancy - Prostate	Y	Y
26	Cancers	Solid organ cancer - primary	Primary Malignancy - Skin	Y	Y
27	Cancers	Solid organ cancer - primary	Primary Malignancy - Stomach	Y	Y
28	Cancers	Solid organ cancer - primary	Primary Malignancy - Testis	Y	Y
29	Cancers	Solid organ cancer - primary	Primary Malignancy - Thyroid	Y	Y
30	Cancers	Solid organ cancer - primary	Primary Malignancy - Uterus	Y	Y
31	Cancers	Solid organ cancer - secondary	Secondary Malignancy - Adrenal	N	Y
32	Cancers	Solid organ cancer - secondary	Secondary Malignancy - Bone	N	Y
33	Cancers	Solid organ cancer - secondary	Secondary Malignancy - Bowel	N	Y
34	Cancers	Solid organ cancer - secondary	Secondary Malignancy - Brain	N	Y
35	Cancers	Solid organ cancer - secondary	Secondary Malignancy - Liver	N	Y
36	Cancers	Solid organ cancer - secondary	Secondary Malignancy - Lung	N	Y
37	Cancers	Solid organ cancer - secondary	Secondary Malignancy - Lymph Nodes	N	Y
38	Cancers	Solid organ cancer - secondary	Secondary Malignancy - other	N	Y
39	Cancers	Solid organ cancer - secondary	Secondary Malignancy - Peritoneum	N	Y
40	Cancers	Solid organ cancer - secondary	Secondary Malignancy - Pleura	N	Y
41	Diseases of the Circulatory System	Cardiac conduction disorder	Atrioventricular Block, first degree	N	Y
42	Diseases of the Circulatory System	Cardiac conduction disorder	Atrioventricular Block, second degree	N	Y
43	Diseases of the Circulatory System	Cardiac conduction disorder	Bifascicular Block	N	Y
44	Diseases of the Circulatory System	Cardiac conduction disorder	Left Bundle Branch Block	N	Y
45	Diseases of the Circulatory System	Cardiac conduction disorder	Right Bundle Branch Block	N	Y
46	Diseases of the Circulatory System	Cardiac conduction disorder	Trifascicular Block	N	Y

Conditions Hirarchy - R Shiny Tool<br>(List of conditions to be supported as index condition (N=128) and comorbidities (N=161) in the R shiny tool)<br>Note: The current version of the r shiny application allows use of individual conditions to be selected as index condition whereas comorbidities can be selected either as body system or as condition group or as individual condition					
47	Diseases of the Circulatory System	Cardiac dysrhythmia	Atrial Fibrillation	Y	Y
48	Diseases of the Circulatory System	Cardiac dysrhythmia	Sick Sinus Syndrome	Y	Y
49	Diseases of the Circulatory System	Cardiac dysrhythmia	Supraventricular Tachycardia	Y	Y
50	Diseases of the Circulatory System	Cardiac dysrhythmia	Ventricular Tachycardia	Y	Y
51	Diseases of the Circulatory System	Cardiomyopathy	Cardiomyopathy other	Y	Y
52	Diseases of the Circulatory System	Cardiomyopathy	Dilated cardiomyopathy	Y	Y
53	Diseases of the Circulatory System	Cardiomyopathy	Hypertrophic cardiomyopathy	Y	Y
54	Diseases of the Circulatory System	Cerebral haemorrhage	Intracerebral Haemorrhage	Y	Y
55	Diseases of the Circulatory System	Cerebral haemorrhage	Subarachnoid Haemorrhage	Y	Y
56	Diseases of the Circulatory System	Coronary heart disease	Coronary Heart Disease (not otherwise specified)	Y	Y
57	Diseases of the Circulatory System	Coronary heart disease	Myocardial Infarction	Y	Y
58	Diseases of the Circulatory System	Coronary heart disease	Stable Angina	Y	Y
59	Diseases of the Circulatory System	Coronary heart disease	Unstable Angina	Y	Y
60	Diseases of the Circulatory System	Heart block	Atrioventricular Block, third degree	Y	Y
61	Diseases of the Circulatory System	Heart failure	Heart Failure	Y	Y
62	Diseases of the Circulatory System	Heart valve disease	Multiple valve disorder	Y	Y
63	Diseases of the Circulatory System	Heart valve disease	Non-rheumatic Aortic valve disorder	Y	Y
64	Diseases of the Circulatory System	Heart valve disease	Non-rheumatic Mitral valve disorder	Y	Y
65	Diseases of the Circulatory System	Heart valve disease	Rheumatic Valve Disorder	Y	Y
66	Diseases of the Circulatory System	Hypertension	Hypertension	Y	Y
67	Diseases of the Circulatory System	Peripheral artery disease	Peripheral Arterial Disease	Y	Y
68	Diseases of the Circulatory System	Primary pulmonary hypertension	Primary Pulmonary Hypertension	Y	Y
69	Diseases of the Circulatory System	Subdural haematoma	Subdural haematoma	N	Y
70	Diseases of the Circulatory System	Thromboembolic and other non-haemorrhagic stroke/TIA	Ischaemic Stroke	Y	Y
71	Diseases of the Circulatory System	Thromboembolic and other non-haemorrhagic stroke/TIA	Stroke not otherwise specified	Y	Y
72	Diseases of the Circulatory System	Thromboembolic and other non-haemorrhagic stroke/TIA	Transient Ischaemic Attack	Y	Y
73	Diseases of the Circulatory System	Venous thromboembolic disease	Pulmonary Embolism	Y	Y
74	Diseases of the Circulatory System	Venous thromboembolic disease	Venous thrombolism (Excl PE)	Y	Y
75	Diseases of the Digestive System	Chronic liver disease	Alcoholic Liver Disease	Y	Y
76	Diseases of the Digestive System	Chronic liver disease	Autoimmune liver disease	Y	Y
77	Diseases of the Digestive System	Chronic liver disease	Chronic Viral Hepatitis	Y	Y
78	Diseases of the Digestive System	Chronic liver disease	Cirrhosis	Y	Y
79	Diseases of the Digestive System	Chronic liver disease	Liver Failure	Y	Y
80	Diseases of the Digestive System	Chronic liver disease	Oesophageal Varices	Y	Y
81	Diseases of the Digestive System	Chronic liver disease	Portal Hypertension	Y	Y
82	Diseases of the Digestive System	Coeliac Disease	Coeliac Disease	Y	Y
83	Diseases of the Digestive System	Fatty Liver	Fatty Liver	Y	Y

Conditions Hirarchy - R Shiny Tool<br>(List of conditions to be supported as index condition (N=128) and comorbidities (N=161) in the R shiny tool)<br>Note: The current version of the r shiny application allows use of individual conditions to be selected as index condition whereas comorbidities can be selected either as body system or as condition group or as individual condition					
84	Diseases of the Digestive System	Inflammatory bowel disease	Crohns Disease	Y	Y
85	Diseases of the Digestive System	Inflammatory bowel disease	Ulcerative Colitis	Y	Y
86	Diseases of the Digestive System	Ulcer and upper GI acid conditions	Barrett's Oesophagus	Y	Y
87	Diseases of the Digestive System	Ulcer and upper GI acid conditions	Diverticular Disease	Y	Y
88	Diseases of the Digestive System	Ulcer and upper GI acid conditions	Gastritis	N	Y
89	Diseases of the Digestive System	Ulcer and upper GI acid conditions	Gastro-oesophageal Reflux Disease	Y	Y
90	Diseases of the Digestive System	Ulcer and upper GI acid conditions	Oesophageal Ulcer	Y	Y
91	Diseases of the Digestive System	Ulcer and upper GI acid conditions	Peptic Ulcer	Y	Y
92	Diseases of the Endocrine System	Diabetes mellitus	Diabetes Mellitus other or not specified	Y	Y
93	Diseases of the Endocrine System	Diabetes mellitus	Diabetic Eye Disease	Y	Y
94	Diseases of the Endocrine System	Diabetes mellitus	Diabetic Neuropathy	Y	Y
95	Diseases of the Endocrine System	Diabetes mellitus	Type 1 Diabetes Mellitus	Y	Y
96	Diseases of the Endocrine System	Diabetes mellitus	Type 2 Diabetes Mellitus	Y	Y
97	Diseases of the Endocrine System	Hyperparathyroidism	Hyperparathyroidism	Y	Y
98	Diseases of the Endocrine System	Obesity	Obesity	Y	Y
99	Diseases of the Endocrine System	Thyroid Disease	Thyroid Disease	Y	Y
100	Diseases of the Genitourinary system	Acute Kidney Injury	Acute Kidney Injury	N	Y
101	Diseases of the Genitourinary system	Benign prostatic hypertrophy	Benign Prostatic Hyperplasia	Y	Y
102	Diseases of the Genitourinary system	Bladder, renal stone, obstruction and reflux	Neuropathic Bladder	N	Y
103	Diseases of the Genitourinary system	Bladder, renal stone, obstruction and reflux	Obstructive and reflux uropathy	N	Y
104	Diseases of the Genitourinary system	Bladder, renal stone, obstruction and reflux	Urinary Incontinence	N	Y
105	Diseases of the Genitourinary system	Bladder, renal stone, obstruction and reflux	Urolithiasis	N	Y
106	Diseases of the Genitourinary system	Chronic renal disease	End Stage Renal Disease	Y	Y
107	Diseases of the Genitourinary system	Chronic renal disease	Glomerulonephritis	Y	Y
108	Diseases of the Genitourinary system	Chronic renal disease	Tubulo-interstitial Nephropathy	Y	Y
109	Diseases of the Genitourinary system	Endometriosis	Endometriosis	Y	Y
110	Diseases of the Genitourinary system	Erectile Dysfunction	Erectile Dysfunction	Y	Y
111	Diseases of the Respiratory System	Chronic lung disease	Asthma	Y	Y
112	Diseases of the Respiratory System	Chronic lung disease	Bronchiectasis	Y	Y
113	Diseases of the Respiratory System	Chronic lung disease	Chronic Obstructive Pulmonary Disease	Y	Y
114	Diseases of the Respiratory System	Chronic lung disease	Cystic Fibrosis	Y	Y
115	Diseases of the Respiratory System	Chronic lung disease	Pulmonary Fibrosis	Y	Y
116	Diseases of the Respiratory System	Chronic lung disease	Respiratory Failure	N	Y
117	Diseases of the Respiratory System	Sleep apnoea	Sleep apnoea	Y	Y
118	Haematological/Immunological conditions	Marrow disorders	Agranulocytosis	N	Y
119	Haematological/Immunological conditions	Marrow disorders	Aplastic Anaemia	Y	Y
120	Haematological/Immunological conditions	Marrow disorders	Primary thrombocytopaenia	Y	Y

Conditions Hirarchy - R Shiny Tool<br>(List of conditions to be supported as index condition (N=128) and comorbidities (N=161) in the R shiny tool)<br>Note: The current version of the r shiny application allows use of individual conditions to be selected as index condition whereas comorbidities can be selected either as body system or as condition group or as individual condition					
121	Haematological/Immunological conditions	Marrow disorders	Sickle Cell Disease	Y	Y
122	Haematological/Immunological conditions	Marrow disorders	Thalassaemia	Y	Y
123	Infectious Diseases	HIV	HIV	Y	Y
124	Mental Health Disorders	Alcohol and substance misuse	Alcohol Misuse	N	Y
125	Mental Health Disorders	Alcohol and substance misuse	Substance Misuse	N	Y
126	Mental Health Disorders	Anxiety	Anxiety	Y	Y
127	Mental Health Disorders	Autism	Autism	Y	Y
128	Mental Health Disorders	Bipolar Affective Disorder	Bipolar Affective Disorder	Y	Y
129	Mental Health Disorders	Delirium	Delirium	N	Y
130	Mental Health Disorders	Dementia	Dementia	Y	Y
131	Mental Health Disorders	Depression	Depression	Y	Y
132	Mental Health Disorders	Eating Disorders	Eating Disorders	Y	Y
133	Mental Health Disorders	Intellectual disability	Down Syndrome	Y	Y
134	Mental Health Disorders	Intellectual disability	Intellectual Disability	Y	Y
135	Mental Health Disorders	Obsessive Compulsive Disorder	Obsessive Compulsive Disorder	Y	Y
136	Mental Health Disorders	Schizophrenia	Schizophrenia	Y	Y
137	Musculoskeletal conditions	Connective tissue disease	Ankylosing Spondylosis	Y	Y
138	Musculoskeletal conditions	Connective tissue disease	Giant Cell Arteritis	Y	Y
139	Musculoskeletal conditions	Connective tissue disease	Juvenile Arthritis	Y	Y
140	Musculoskeletal conditions	Connective tissue disease	Lupus Erythematosus	Y	Y
141	Musculoskeletal conditions	Connective tissue disease	Polymyalgia Rheumatica	Y	Y
142	Musculoskeletal conditions	Connective tissue disease	Psoriatic Arthritis	Y	Y
143	Musculoskeletal conditions	Connective tissue disease	Raynauds Disease	N	Y
144	Musculoskeletal conditions	Connective tissue disease	Reactive Arthritis	Y	Y
145	Musculoskeletal conditions	Connective tissue disease	Rheumatoid Arthritis	Y	Y
146	Musculoskeletal conditions	Connective tissue disease	Scleroderma	Y	Y
147	Musculoskeletal conditions	Connective tissue disease	Sjogren Syndrome	Y	Y
148	Musculoskeletal conditions	Gout	Gout	Y	Y
149	Musculoskeletal conditions	Osteoarthritis	Osteoarthritis	Y	Y
150	Musculoskeletal conditions	Osteoporosis and low impact fracture	Collapsed Vertebra	N	Y
151	Musculoskeletal conditions	Osteoporosis and low impact fracture	Fracture Hip	N	Y
152	Musculoskeletal conditions	Osteoporosis and low impact fracture	Fracture Wrist	N	Y
153	Musculoskeletal conditions	Osteoporosis and low impact fracture	Osteoporosis	N	Y
154	Neurological conditions	Cerebral Palsy	Cerebral Palsy	Y	Y
155	Neurological conditions	Epilepsy	Epilepsy	Y	Y
156	Neurological conditions	Motor Neurone Disease	Motor Neurone Disease	Y	Y
157	Neurological conditions	Multiple Sclerosis	Multiple Sclerosis	Y	Y
158	Neurological conditions	Myasthenia Gravis	Myasthenia Gravis	Y	Y
159	Neurological conditions	Parkinson's Disease	Parkinson's Disease	Y	Y
160	Neurological conditions	Peripheral Neuropathy	Peripheral Neuropathy	Y	Y
161	Skin conditions	Psoriasis	Psoriasis	Y	Y
