# Supplementary material for: A Digital Tool for Clinical Evidence–Driven Guideline Development by Studying Properties of Trial Eligible and Ineligible Populations: Development and Usability Study
Source: J Med Internet Res. 2025 Jan 16;27:e52385. doi: 10.2196/52385 (PMC11783027; doi:10.2196/52385)
Supplement: Multimedia Appendix 4 [file jmir_v27i1e52385_app4.pdf]

Index Condition Analysis Report

Shahzad Mumtaz

25-07-2023

Index Condition Analysis - Gout

This report focuses on the aggregate level statistical information generated from the Clinical Primary Care Data (CPRD)-Gold data. This analysis includes all the patients who has condition diagnosed for the first time on or before a specified date. Following is the summary statistics.

- Total number of patients 33480
- Index condition date 11/30/2015 (All patients registered on or before the specified date with Gout)

The report presents the following tables:

- Demography infomration
- electronic Frailty Index (eFI) distribution
- Charlson score distribution
- Comorbidities (Top 10).
    - Comorbidities Body System (Top 10)
    - Comorbidities Condition Group (Top 10)
    - Comorbidities Individual Condition (Top 10)
- Comorbidities (User Selected).
    - Comorbidities Condition Group (User Selected)
- Drugs (Top 10).
    - Drug Chapter (Top 10)
    - Drug Class (Top 10)
    - Drug Name (Top 10)
- Drugs (User Selected).
    - Drug Class (User Selected)
- Outcome
    - Deaths
    - Death Rate
    - Hospital Admissions
    - Hospital Admission Rate

Demography infomration - including age-sex distribution, Ethnicity distribution ,IMD Quintile and Time Since Diagnosis

Table 1: Demography analysis for Index Condition 'Gout' (Index Condition Date: <= 30/11/2015) - (Age-Gender, Ethnicity-Gender, IMD Quintile-gender and Time Since Diagnosis-gender distributions). Note: For disclosure control, all numbers presented are rounded to 10, percentages are rounded to discrete numbers, and percentages presented are column percentages and so totals will not add exactly.

Criteria	All No(%)	Men No(%)	Women No(%)
**All**			
All	33480	26440 (79%)	7040 (21%)
**Age**			
<25	50 (0%)	40 (0%)	20 (0%)
25-34	470 (1%)	410 (2%)	60 (1%)
35-44	1960 (6%)	1780 (7%)	180 (3%)
45-54	4900 (15%)	4390 (17%)	510 (7%)
55-64	6930 (21%)	5880 (22%)	1050 (15%)
65-74	8800 (26%)	7060 (27%)	1740 (25%)
75-84	7210 (22%)	5040 (19%)	2180 (31%)
>=85	3160 (9%)	1860 (7%)	1310 (19%)
**Ethnicity**			
White	27100 (81%)	20860 (79%)	6250 (89%)
Asian	610 (2%)	470 (2%)	140 (2%)
Black	320 (1%)	240 (1%)	80 (1%)
Chinese or Mixed Or Other	360 (1%)	290 (1%)	70 (1%)
Missing	5090 (15%)	4590 (17%)	500 (7%)
**IMD Quintile**			
Q1 (least deprived)	8720 (26%)	7130 (27%)	1590 (23%)
Q2	7400 (22%)	5890 (22%)	1510 (21%)
Q3	7300 (22%)	5710 (22%)	1580 (22%)
Q4	5680 (17%)	4330 (16%)	1350 (19%)
Q5 (most deprived)	4380 (13%)	3380 (13%)	1010 (14%)

Criteria	All No(%)	Men No(%)	Women No(%)
**Time Since Diagnosis**			
>=1 year	31210 (93%)	24790 (94%)	6420 (91%)
< 1 year	2270 (7%)	1650 (6%)	620 (9%)

electronic Frailty Index (eFI)

Table 2: electronic Frailty Index (eFI) for index condition 'Gout' cohort (Index Condition Date: <= 30/11/2015) - age, sex, ethnicity, and IMD quintile distributions. Note: These scores are not validated for under 65s. For disclosure control, all numbers presented are rounded to 10, percentages are rounded to discrete numbers, and percentages presented are row percentages so totals will not add exactly.

Criteria	Fit	Mild Frailty	Moderate Frailty	Severe Frailty
**All**				
All	13650(41%)	10220(31%)	6210(19%)	3400(10%)
**Age**				
<25	40 (85%)	10 (15%)	0 (0%)	0 (0%)
25-34	410 (89%)	50 (10%)	0 (1%)	0 (0%)
35-44	1700 (87%)	210 (11%)	40 (2%)	0 (0%)
45-54	3670 (75%)	1020 (21%)	170 (3%)	40 (1%)
55-64	3850 (56%)	2270 (33%)	660 (10%)	150 (2%)
65-74	2890 (33%)	3540 (40%)	1790 (20%)	580 (7%)
75-84	940 (13%)	2420 (33%)	2370 (33%)	1490 (21%)
>=85	140 (4%)	700 (22%)	1180 (37%)	1140 (36%)
**Sex**				
Men	12310 (47%)	8040 (30%)	4220 (16%)	1870 (7%)
Women	1330 (19%)	2190 (31%)	1990 (28%)	1530 (22%)
**Ethnicity**				
White	9100 (34%)	8890 (33%)	5850 (22%)	3260 (12%)
Asian	230 (37%)	200 (32%)	120 (20%)	70 (11%)
Black	120 (36%)	100 (33%)	60 (20%)	30 (11%)
Chinese or Mixed Or Other	160 (46%)	120 (33%)	50 (14%)	20 (7%)
Missing	4040 (79%)	910 (18%)	130 (3%)	20 (0%)
**IMD Quintile**				
Q1 (least deprived)	4050 (46%)	2570 (29%)	1420 (16%)	690 (8%)
Q2	3100 (42%)	2240 (30%)	1330 (18%)	730 (10%)
Q3	2860 (39%)	2280 (31%)	1430 (20%)	730 (10%)
Q4	2100 (37%)	1760 (31%)	1140 (20%)	670 (12%)

Criteria	Fit	Mild Frailty	Moderate Frailty	Severe Frailty
Q5 (most deprived)	1540 (35%)	1370 (31%)	890 (20%)	580 (13%)

Charlson Comorbidities Score

Table 3: Charlson Comorbidity Score for index condition 'Gout'(Index Condition Date: <= 30/11/2015) - age, sex, ethnicity, and IMD quintile distributions. Note: For disclosure control, all numbers presented are rounded to 10, percentages are rounded to discrete numbers and percentages presented are row percentages. So totals will not add exactly.

Criteria	Score 0	Score 1	Score 2	Score 3+
**All**				
All	11500(34%)	6070(18%)	5590(17%)	10320(31%)
**Age**				
<25	30 (62%)	20 (31%)	0 (2%)	0 (6%)
25-34	300 (64%)	130 (27%)	20 (5%)	20 (3%)
35-44	1310 (67%)	460 (23%)	120 (6%)	70 (4%)
45-54	2970 (61%)	1080 (22%)	520 (11%)	330 (7%)
55-64	3210 (46%)	1580 (23%)	1150 (17%)	990 (14%)
65-74	2520 (29%)	1690 (19%)	1780 (20%)	2810 (32%)
75-84	970 (13%)	880 (12%)	1430 (20%)	3940 (55%)
>=85	200 (6%)	250 (8%)	560 (18%)	2160 (68%)
**Sex**				
Men	10080 (38%)	4930 (19%)	4260 (16%)	7170 (27%)
Women	1420 (20%)	1140 (16%)	1330 (19%)	3150 (45%)
**Ethnicity**				
White	7880 (29%)	4850 (18%)	4760 (18%)	9610 (35%)
Asian	180 (29%)	120 (19%)	120 (19%)	200 (32%)
Black	90 (28%)	60 (18%)	60 (18%)	120 (36%)
Chinese or Mixed Or Other	140 (38%)	70 (19%)	70 (19%)	90 (24%)
Missing	3210 (63%)	980 (19%)	590 (12%)	310 (6%)
**IMD Quintile**				
Q1 (least deprived)	3280 (38%)	1490 (17%)	1490 (17%)	2470 (28%)
Q2	2600 (35%)	1330 (18%)	1220 (17%)	2250 (30%)
Q3	2410 (33%)	1390 (19%)	1220 (17%)	2280 (31%)
Q4	1840 (32%)	1000 (18%)	970 (17%)	1860 (33%)
Q5 (most deprived)	1370 (31%)	870 (20%)	680 (16%)	1460 (33%)

Comorbidities (Top 10)

Body System Comorbidities (Top 10)

Table 4a: Top 10 Comorbities (1 to 5) - Body System comorbidites for index condition 'Gout' (Index Condition Date: <= 30/11/2015) - age, sex, ethnicity, and IMD quintile distributions. Note: For disclosure control, all numbers presented are rounded to 10, percentages are rounded to discrete numbers, and percentages presented are row percentages and so totals will not add exactly.

	Diseases of the Circulatory System	Musculoskeletal conditions	Diseases of the Genitourinary system	Diseases of the Digestive System	Diseases of the Endocrine System
**All**					
All	22730 (68%)	13920 (42%)	13140 (39%)	12820 (38%)	11980 (36%)
**Age**					
<25	0 (8%)	10 (15%)	0 (8%)	0 (10%)	0 (6%)
25-34	50 (10%)	50 (11%)	50 (10%)	70 (15%)	60 (14%)
35-44	380 (19%)	190 (10%)	220 (11%)	370 (19%)	340 (17%)
45-54	1820 (37%)	930 (19%)	1050 (21%)	1200 (25%)	1150 (23%)
55-64	4180 (60%)	2260 (33%)	2340 (34%)	2290 (33%)	2310 (33%)
65-74	6840 (78%)	4040 (46%)	3960 (45%)	3660 (42%)	3540 (40%)
75-84	6480 (90%)	4270 (59%)	3790 (53%)	3590 (50%)	3260 (45%)
>=85	2980 (94%)	2180 (69%)	1720 (55%)	1620 (51%)	1310 (41%)
**Sex**					
Men	17180 (65%)	9500 (36%)	10920 (41%)	9480 (36%)	8400 (32%)
Women	5550 (79%)	4420 (63%)	2220 (32%)	3340 (47%)	3590 (51%)
**Ethnicity**					
White	19820 (73%)	12670 (47%)	11680 (43%)	11680 (43%)	10410 (38%)
Asian	420 (68%)	220 (36%)	270 (44%)	250 (41%)	290 (47%)
Black	240 (74%)	110 (34%)	150 (46%)	120 (37%)	160 (49%)
Chinese or Mixed or Other	240 (68%)	110 (30%)	150 (42%)	110 (32%)	120 (35%)
Missing	2010 (39%)	810 (16%)	890 (18%)	660 (13%)	1010 (20%)
**IMD Quintile**					

	Diseases of the Circulatory System	Musculoskeletal conditions	Diseases of the Genitourinary system	Diseases of the Digestive System	Diseases of the Endocrine System
Q1 (least deprived	5690 (65%)	3280 (38%)	3400 (39%)	3180 (36%)	2680 (31%)
Q2	4990 (67%)	3050 (41%)	2930 (40%)	2740 (37%)	2560 (35%)
Q3	5040 (69%)	3170 (43%)	2850 (39%)	2780 (38%)	2690 (37%)
Q4	3930 (69%)	2480 (44%)	2230 (39%)	2240 (39%)	2240 (39%)
Q5 (most deprived)	3070 (70%)	1950 (44%)	1730 (40%)	1890 (43%)	1800 (41%)

Table 4b: Top 10 Comorbities (6 to 10) - Body System comorbidities for index condition 'Gout' (Index Condition Date: <= 30/11/2015) - age, sex, ethnicity, and IMD quintile distributions. Note: For disclosure control, all numbers presented are rounded to 10, percentages are rounded to discrete numbers, and percentages presented are row percentages and so totals will not add exactly.

	Mental Health Disorders	Diseases of the Respiratory System	Cancers	Neurological conditions	Skin conditions
**All**					
All	11440 (34%)	7990 (24%)	6120 (18%)	2990 (9%)	2200 (7%)
**Age**					
<25	20 (33%)	20 (37%)	0 (6%)	0 (4%)	0 (6%)
25-34	150 (32%)	110 (24%)	10 (2%)	20 (4%)	20 (5%)
35-44	650 (33%)	400 (21%)	30 (2%)	100 (5%)	110 (5%)
45-54	1680 (34%)	970 (20%)	190 (4%)	310 (6%)	280 (6%)
55-64	2510 (36%)	1460 (21%)	640 (9%)	550 (8%)	420 (6%)
65-74	2960 (34%)	2200 (25%)	1720 (20%)	860 (10%)	680 (8%)
75-84	2350 (33%)	1980 (27%)	2290 (32%)	820 (11%)	470 (7%)
>=85	1130 (36%)	850 (27%)	1220 (38%)	320 (10%)	220 (7%)
**Sex**					
Men	8240 (31%)	5890 (22%)	4550 (17%)	2300 (9%)	1700 (6%)
Women	3210 (46%)	2100 (30%)	1560 (22%)	680 (10%)	500 (7%)
**Ethnicity**					
White	9990 (37%)	7030 (26%)	5770 (21%)	2700 (10%)	1920 (7%)
Asian	160 (27%)	170 (27%)	50 (9%)	60 (9%)	30 (5%)
Black	90 (27%)	80 (24%)	40 (12%)	30 (8%)	10 (2%)
Chinese or Mixed or Other	90 (24%)	60 (18%)	40 (10%)	30 (8%)	20 (5%)
Missing	1120 (22%)	660 (13%)	220 (4%)	170 (3%)	220 (4%)
**IMD Quintile**					

	Mental Health Disorders	Diseases of the Respiratory System	Cancers	Neurological conditions	Skin conditions
Q1 (least deprived)	2580 (30%)	1810 (21%)	1680 (19%)	710 (8%)	580 (7%)
Q2	2340 (32%)	1690 (23%)	1440 (20%)	660 (9%)	440 (6%)
Q3	2420 (33%)	1730 (24%)	1360 (19%)	640 (9%)	470 (6%)
Q4	2140 (38%)	1500 (26%)	980 (17%)	530 (9%)	410 (7%)
Q5 (most deprived)	1960 (45%)	1260 (29%)	650 (15%)	450 (10%)	300 (7%)

Condition Group Comorbidities (Top 10)

Table 5a: Top 10 Comorbities (1 to 5) - Condition group comorbidities for index condition 'Gout' (Index Condition Date: <= 30/11/2015) - age, sex, ethnicity, and IMD quintile distributions. Note: For disclosure control, all numbers presented are rounded to 10, percentages are rounded to discrete numbers, and percentages presented are row percentages and so totals will not add exactly.

	Hypertension	Ulcer and upper GI acid conditions	Osteoarthritis	Chronic lung disease	Depression
**All**					
All	20540 (61%)	12120 (36%)	11530 (34%)	7260 (22%)	7050 (21%)
**Age**					
<25	0 (4%)	0 (8%)	0 (0%)	20 (35%)	10 (19%)
25-34	30 (6%)	60 (13%)	10 (2%)	110 (24%)	100 (21%)
35-44	300 (16%)	340 (17%)	80 (4%)	360 (18%)	430 (22%)
45-54	1590 (32%)	1090 (22%)	610 (12%)	840 (17%)	1100 (23%)
55-64	3710 (54%)	2100 (30%)	1810 (26%)	1270 (18%)	1660 (24%)
65-74	6210 (71%)	3470 (39%)	3420 (39%)	1970 (22%)	1840 (21%)
75-84	5940 (82%)	3470 (48%)	3720 (52%)	1880 (26%)	1330 (18%)
>=85	2760 (87%)	1580 (50%)	1870 (59%)	830 (26%)	570 (18%)
**Sex**					
Men	15360 (58%)	8920 (34%)	7860 (30%)	5230 (20%)	4810 (18%)
Women	5190 (74%)	3190 (45%)	3670 (52%)	2030 (29%)	2240 (32%)
**Ethnicity**					

	Hypertension	Ulcer and upper GI acid conditions	Osteoarthritis	Chronic lung disease	Depression
White	17850 (66%)	11080 (41%)	10580 (39%)	6380 (24%)	6200 (23%)
Asian	380 (62%)	230 (37%)	180 (30%)	150 (24%)	110 (18%)
Black	230 (72%)	100 (33%)	100 (30%)	70 (21%)	60 (17%)
Chinese or Mixed or Other	210 (58%)	100 (29%)	80 (24%)	60 (16%)	60 (17%)
Missing	1880 (37%)	600 (12%)	590 (12%)	610 (12%)	620 (12%)
**IMD Quintile**					
Q1 (least deprived)	5100 (58%)	2990 (34%)	2700 (31%)	1630 (19%)	1480 (17%)
Q2	4500 (61%)	2590 (35%)	2490 (34%)	1510 (20%)	1400 (19%)
Q3	4560 (62%)	2640 (36%)	2620 (36%)	1570 (22%)	1550 (21%)
Q4	3590 (63%)	2110 (37%)	2080 (37%)	1370 (24%)	1350 (24%)
Q5 (most deprived)	2800 (64%)	1790 (41%)	1640 (37%)	1180 (27%)	1270 (29%)

Table 5b: Top 10 Comorbities (6 to 10) - Condition group comorbidties for index condition 'Gout' (Index Condition Date: <= 30/11/2015) - age, sex, ethnicity, and IMD quintile distributions. Note: For disclosure control, all numbers presented are rounded to 10, percentages are rounded to discrete numbers, and percentages presented are row percentages and so totals will not add exactly.

	Coronary heart disease	Diabetes mellitus	Erectile Dysfunction	Obesity	Solid organ cancer - primary
**All**					
All	6980 (21%)	6650 (20%)	6030 (18%)	5790 (17%)	5690 (17%)
**Age**					
<25	0 (0%)	0 (0%)	0 (0%)	0 (6%)	0 (2%)
25-34	0 (1%)	10 (2%)	10 (3%)	50 (11%)	0 (1%)
35-44	30 (2%)	110 (6%)	90 (5%)	230 (12%)	20 (1%)
45-54	240 (5%)	500 (10%)	520 (11%)	710 (15%)	160 (3%)
55-64	800 (12%)	1200 (17%)	1400 (20%)	1310 (19%)	580 (8%)
65-74	2050 (23%)	2110 (24%)	2240 (26%)	1750 (20%)	1590 (18%)
75-84	2560 (35%)	1990 (28%)	1420 (20%)	1370 (19%)	2160 (30%)
>=85	1300 (41%)	730 (23%)	340 (11%)	360 (11%)	1170 (37%)
**Sex**					
Men	5470 (21%)	4910 (19%)	6030 (23%)	4080 (15%)	4220 (16%)
Women	1520 (22%)	1740 (25%)	0 (0%)	1710 (24%)	1470 (21%)
**Ethnicity**					
White	6560 (24%)	5700 (21%)	5160 (19%)	5140 (19%)	5380 (20%)
Asian	160 (27%)	220 (36%)	130 (21%)	100 (17%)	40 (7%)
Black	60 (17%)	100 (32%)	80 (25%)	70 (22%)	30 (9%)
Chinese or Mixed or Other	70 (20%)	90 (26%)	70 (19%)	50 (14%)	30 (9%)
Missing	140 (3%)	540 (11%)	590 (12%)	420 (8%)	200 (4%)
**IMD Quintile**					

	Coronary heart disease	Diabetes mellitus	Erectile Dysfunction	Obesity	Solid organ cancer - primary
Q1 (least deprived)	1630 (19%)	1470 (17%)	1560 (18%)	1170 (13%)	1580 (18%)
Q2	1470 (20%)	1420 (19%)	1340 (18%)	1160 (16%)	1340 (18%)
Q3	1600 (22%)	1470 (20%)	1320 (18%)	1320 (18%)	1260 (17%)
Q4	1240 (22%)	1250 (22%)	980 (17%)	1160 (20%)	910 (16%)
Q5 (most deprived)	1040 (24%)	1040 (24%)	830 (19%)	980 (22%)	600 (14%)

Individual Condition Comorbidities (Top 10)

Table 6a: Top 10 Comorbities (1 to 5) - Individual condition comorbidities for index condition 'Gout' (Index Condition Date: <= 30/11/2015) - age, sex, ethnicity, and IMD quintile distributions. Note: For disclosure control, all numbers presented are rounded to 10, percentages are rounded to discrete numbers, and percentages presented are row percentages and so totals will not add exactly.

	Hypertension	Osteoarthritis	Depression	Type 2 Diabetes Mellitus	Coronary Heart Disease (not otherwise specified)
**All**					
All	20540 (61%)	11530 (34%)	7050 (21%)	6460 (19%)	6090 (18%)
**Age**					
<25	0 (4%)	0 (0%)	10 (19%)	0 (0%)	0 (0%)
25-34	30 (6%)	10 (2%)	100 (21%)	0 (1%)	0 (0%)
35-44	300 (16%)	80 (4%)	430 (22%)	90 (5%)	20 (1%)
45-54	1590 (32%)	610 (12%)	1100 (23%)	480 (10%)	190 (4%)
55-64	3710 (54%)	1810 (26%)	1660 (24%)	1170 (17%)	650 (9%)
65-74	6210 (71%)	3420 (39%)	1840 (21%)	2060 (23%)	1790 (20%)
75-84	5940 (82%)	3720 (52%)	1330 (18%)	1940 (27%)	2300 (32%)
>=85	2760 (87%)	1870 (59%)	570 (18%)	710 (23%)	1140 (36%)
**Sex**					
Men	15360 (58%)	7860 (30%)	4810 (18%)	4770 (18%)	4860 (18%)
Women	5190 (74%)	3670 (52%)	2240 (32%)	1690 (24%)	1230 (17%)
**Ethnicity**					
White	17850 (66%)	10580 (39%)	6200 (23%)	5520 (20%)	5730 (21%)
Asian	380 (62%)	180 (30%)	110 (18%)	210 (35%)	140 (23%)
Black	230 (72%)	100 (30%)	60 (17%)	100 (31%)	40 (14%)
Chinese or Mixed or Other	210 (58%)	80 (24%)	60 (17%)	90 (25%)	60 (17%)
Missing	1880 (37%)	590 (12%)	620 (12%)	540 (11%)	110 (2%)
**IMD Quintile**					
Q1 (least deprived)	5100 (58%)	2700 (31%)	1480 (17%)	1440 (16%)	1440 (17%)
Q2	4500 (61%)	2490 (34%)	1400 (19%)	1370 (19%)	1280 (17%)

	Hypertension	Osteoarthritis	Depression	Type 2 Diabetes Mellitus	Coronary Heart Disease (not otherwise specified)
Q3	4560 (62%)	2620 (36%)	1550 (21%)	1430 (20%)	1380 (19%)
Q4	3590 (63%)	2080 (37%)	1350 (24%)	1210 (21%)	1080 (19%)
Q5 (most deprived)	2800 (64%)	1640 (37%)	1270 (29%)	1010 (23%)	910 (21%)

Table 6b: Top 10 Comorbities (6 to 10) - Condition group comorbidities for index condition 'Gout' (Index Condition Date: <= 30/11/2015) - age, sex, ethnicity, and IMD quintile distributions. Note: For disclosure control, all numbers presented are rounded to 10, percentages are rounded to discrete numbers, and percentages presented are row percentages and so totals will not add exactly.

	Erectile Dysfunction	Gastro-oesophageal Reflux Disease	Obesity	Asthma	Anxiety
**All**					
All	6030 (18%)	5950 (18%)	5790 (17%)	5460 (16%)	5280 (16%)
**Age**					
<25	0 (0%)	0 (2%)	0 (6%)	20 (35%)	10 (19%)
25-34	10 (3%)	40 (8%)	50 (11%)	110 (23%)	70 (15%)
35-44	90 (5%)	210 (11%)	230 (12%)	350 (18%)	320 (16%)
45-54	520 (11%)	640 (13%)	710 (15%)	770 (16%)	780 (16%)
55-64	1400 (20%)	1180 (17%)	1310 (19%)	1060 (15%)	1230 (18%)
65-74	2240 (26%)	1660 (19%)	1750 (20%)	1400 (16%)	1440 (16%)
75-84	1420 (20%)	1580 (22%)	1370 (19%)	1240 (17%)	1010 (14%)
>=85	340 (11%)	630 (20%)	360 (11%)	520 (16%)	420 (13%)
**Sex**					
Men	6030 (23%)	4350 (16%)	4080 (15%)	3860 (15%)	3540 (13%)
Women	0 (0%)	1600 (23%)	1710 (24%)	1590 (23%)	1740 (25%)
**Ethnicity**					
White	5160 (19%)	5330 (20%)	5140 (19%)	4660 (17%)	4620 (17%)
Asian	130 (21%)	140 (23%)	100 (17%)	120 (20%)	60 (10%)
Black	80 (25%)	60 (18%)	70 (22%)	60 (18%)	30 (11%)
Chinese or Mixed or Other	70 (19%)	50 (15%)	50 (14%)	40 (12%)	40 (10%)
Missing	590 (12%)	360 (7%)	420 (8%)	570 (11%)	530 (10%)
**IMD Quintile**					

	Erectile Dysfunction	Gastro-oesophageal Reflux Disease	Obesity	Asthma	Anxiety
Q1 (least deprived)	1560 (18%)	1480 (17%)	1170 (13%)	1290 (15%)	1220 (14%)
Q2	1340 (18%)	1270 (17%)	1160 (16%)	1140 (15%)	1100 (15%)
Q3	1320 (18%)	1290 (18%)	1320 (18%)	1160 (16%)	1090 (15%)
Q4	980 (17%)	1020 (18%)	1160 (20%)	1000 (18%)	940 (17%)
Q5 (most deprived)	830 (19%)	890 (20%)	980 (22%)	850 (19%)	930 (21%)

Condition Group Comorbidities (User selected)

Table 7 : User selected Condition Group comorbidites (1 to 4) for index condition 'params$indexConditionName' (Index Condition Date: <= 30/11/2015) - age, sex, ethnicity, and IMD quintile distributions. Note: For disclosure control, all numbers presented are rounded to 10, percentages are rounded to discrete numbers, and percentages presented are row percentages and so totals will not add exactly.

	Coronary heart disease	Hypertension	Ulcer and upper GI acid conditions	Chronic lung disease
**All**				
All	6980 (21%)	20540 (61%)	12120 (36%)	7260 (22%)
**Age**				
<25	0 (0%)	0 (4%)	0 (8%)	20 (35%)
25-34	0 (1%)	30 (6%)	60 (13%)	110 (24%)
35-44	30 (2%)	300 (16%)	340 (17%)	360 (18%)
45-54	240 (5%)	1590 (32%)	1090 (22%)	840 (17%)
55-64	800 (12%)	3710 (54%)	2100 (30%)	1270 (18%)
65-74	2050 (23%)	6210 (71%)	3470 (39%)	1970 (22%)
75-84	2560 (35%)	5940 (82%)	3470 (48%)	1880 (26%)
>=85	1300 (41%)	2760 (87%)	1580 (50%)	830 (26%)
**Sex**				
Men	5470 (21%)	15360 (58%)	8920 (34%)	5230 (20%)
Women	1520 (22%)	5190 (74%)	3190 (45%)	2030 (29%)
**Ethnicity**				

	Coronary heart disease	Hypertension	Ulcer and upper GI acid conditions	Chronic lung disease
White	6560 (24%)	17850 (66%)	11080 (41%)	6380 (24%)
Asian	160 (27%)	380 (62%)	230 (37%)	150 (24%)
Black	60 (17%)	230 (72%)	100 (33%)	70 (21%)
Chinese or Mixed or Other	70 (20%)	210 (58%)	100 (29%)	60 (16%)
Missing	140 (3%)	1880 (37%)	600 (12%)	610 (12%)
**IMD Quintile**				
Q1 (least deprived)	1630 (19%)	5100 (58%)	2990 (34%)	1630 (19%)
Q2	1470 (20%)	4500 (61%)	2590 (35%)	1510 (20%)
Q3	1600 (22%)	4560 (62%)	2640 (36%)	1570 (22%)
Q4	1240 (22%)	3590 (63%)	2110 (37%)	1370 (24%)
Q5 (most deprived)	1040 (24%)	2800 (64%)	1790 (41%)	1180 (27%)

Drugs (Top 10)

Drugs Chapter (Top 10)

Table 8a: Top 10 drugs chapter (1 to 5) for index condition 'Gout' (Index Condition Date: <= 30/11/2015) - age, sex, ethnicity, and IMD quintile distributions. Note: For disclosure control, all numbers presented are rounded to 10, percentages are rounded to discrete numbers and percentages presented are row percentages. So totals will not add exactly.

	02 Cardiovascular System	10 Musculoskeletal and Joint Disease	01 Gastro-Intestinal System	04 Central Nervous System	06 Endocrine System
**All**					
All	22310 (67%)	14920 (45%)	11980 (36%)	11720 (35%)	8980 (27%)
**Age**					
<25	0 (4%)	10 (19%)	0 (8%)	10 (19%)	0 (4%)
25-34	40 (9%)	120 (26%)	50 (10%)	70 (14%)	30 (6%)
35-44	330 (17%)	630 (32%)	260 (13%)	380 (19%)	160 (8%)
45-54	1660 (34%)	1880 (38%)	1000 (20%)	990 (20%)	660 (13%)
55-64	4080 (59%)	3070 (44%)	2010 (29%)	2000 (29%)	1490 (22%)
65-74	6960 (79%)	4320 (49%)	3370 (38%)	3110 (35%)	2590 (29%)
75-84	6390 (89%)	3510 (49%)	3540 (49%)	3430 (48%)	2760 (38%)
>=85	2840 (90%)	1380 (44%)	1740 (55%)	1740 (55%)	1300 (41%)
**Sex**					
Men	16900 (64%)	12030 (45%)	8690 (33%)	7820 (30%)	5960 (23%)
Women	5410 (77%)	2900 (41%)	3290 (47%)	3890 (55%)	3020 (43%)
**Ethnicity**					
White	19370 (71%)	12550 (46%)	10930 (40%)	10660 (39%)	7940 (29%)
Asian	400 (65%)	270 (44%)	250 (40%)	240 (39%)	230 (38%)
Black	220 (70%)	140 (43%)	110 (33%)	110 (34%)	110 (34%)
Chinese or Mixed or Other	240 (66%)	170 (48%)	100 (29%)	100 (28%)	100 (28%)
Missing	2080 (41%)	1790 (35%)	590 (12%)	620 (12%)	600 (12%)
**IMD Quintile**					
Q1 (least deprived)	5560 (64%)	3660 (42%)	2710 (31%)	2390 (27%)	2000 (23%)

	02 Cardiovascular System	10 Musculoskeletal and Joint Disease	01 Gastro-Intestinal System	04 Central Nervous System	06 Endocrine System
Q2	4910 (66%)	3270 (44%)	2500 (34%)	2440 (33%)	1980 (27%)
Q3	4950 (68%)	3260 (45%)	2740 (38%)	2550 (35%)	2060 (28%)
Q4	3900 (69%)	2670 (47%)	2230 (39%)	2280 (40%)	1620 (29%)
Q5 (most deprived)	2990 (68%)	2070 (47%)	1800 (41%)	2060 (47%)	1320 (30%)

Table 8b: Top 10 drugs chapter (6 to 10) for index condition 'Gout' (Index Condition Date: <= 30/11/2015) - age, sex, ethnicity, and IMD quintile distributions. Note: For disclosure control, all numbers presented are rounded to 10, percentages are rounded to discrete numbers and percentages presented are row percentages. So totals will not add exactly.

	03 Resipiratory System	05 Infections	09 Nutrition and Blood	07 Obsetrics, Gynaecology and Urinary-Tract Disorders	11 Eye
**All**					
All	5220 (16%)	4710 (14%)	4700 (14%)	3790 (11%)	2960 (9%)
**Age**					
<25	0 (8%)	10 (19%)	0 (4%)	0 (10%)	0 (0%)
25-34	30 (6%)	40 (9%)	20 (3%)	10 (3%)	0 (1%)
35-44	160 (8%)	140 (7%)	80 (4%)	40 (2%)	30 (1%)
45-54	480 (10%)	400 (8%)	280 (6%)	230 (5%)	120 (2%)
55-64	860 (12%)	770 (11%)	600 (9%)	640 (9%)	310 (5%)
65-74	1620 (18%)	1270 (14%)	1130 (13%)	1240 (14%)	790 (9%)
75-84	1470 (20%)	1340 (19%)	1610 (22%)	1150 (16%)	1030 (14%)
>=85	610 (19%)	740 (23%)	990 (31%)	480 (15%)	670 (21%)
**Sex**					
Men	3660 (14%)	3180 (12%)	2770 (10%)	3440 (13%)	1960 (7%)
Women	1570 (22%)	1530 (22%)	1930 (27%)	350 (5%)	1000 (14%)
**Ethnicity**					
White	4650 (17%)	4250 (16%)	4320 (16%)	3390 (13%)	2640 (10%)
Asian	120 (20%)	90 (15%)	150 (25%)	80 (14%)	90 (15%)
Black	60 (18%)	30 (10%)	60 (18%)	40 (12%)	50 (17%)
Chinese or Mixed or Other	60 (16%)	40 (11%)	50 (14%)	40 (11%)	40 (11%)

	03 Resipiratory System	05 Infections	09 Nutrition and Blood	07 Obsetrics, Gynaecology and Urinary-Tract Disorders	11 Eye
Missing	340 (7%)	290 (6%)	120 (2%)	230 (5%)	130 (3%)
**IMD Quintile**					
Q1 (least deprived)	1080 (12%)	1090 (13%)	960 (11%)	990 (11%)	740 (8%)
Q2	1060 (14%)	980 (13%)	1010 (14%)	850 (11%)	640 (9%)
Q3	1150 (16%)	1070 (15%)	1060 (14%)	840 (12%)	660 (9%)
Q4	1040 (18%)	860 (15%)	890 (16%)	610 (11%)	520 (9%)
Q5 (most deprived)	890 (20%)	700 (16%)	780 (18%)	490 (11%)	410 (9%)

Drugs Class (Top 10)

Table 9a: Top 10 drugs class (1 to 5) for index condition 'Gout' (Index Condition Date: <= 30/11/2015) - age, sex, ethnicity, and IMD quintile distributions. Note: For disclosure control, all numbers presented are rounded to 10, percentages are rounded to discrete numbers and percentages presented are row percentages. So totals will not add exactly.

	Statins	Urate lowering therapy	ACE inhibitors	Proton pump inhibitors	Calcium channel blockers
**All**					
All	13740 (41%)	10650 (32%)	9850 (29%)	9580 (29%)	8540 (26%)
**Age**					
<25	0 (0%)	10 (13%)	0 (0%)	0 (8%)	0 (0%)
25-34	10 (3%)	70 (15%)	20 (4%)	40 (8%)	0 (1%)
35-44	120 (6%)	450 (23%)	170 (9%)	230 (12%)	100 (5%)
45-54	820 (17%)	1340 (27%)	860 (18%)	850 (17%)	550 (11%)
55-64	2430 (35%)	2190 (32%)	1950 (28%)	1730 (25%)	1640 (24%)
65-74	4740 (54%)	3070 (35%)	3080 (35%)	2750 (31%)	2820 (32%)
75-84	4120 (57%)	2560 (35%)	2700 (37%)	2740 (38%)	2440 (34%)
>=85	1500 (48%)	970 (31%)	1070 (34%)	1240 (39%)	1000 (32%)
**Sex**					
Men	10812 (41%)	8918 (34%)	7826 (30%)	7024 (27%)	6710 (25%)
Women	2931 (42%)	1730 (25%)	2023 (29%)	2561 (36%)	1834 (26%)
**Ethnicity**					
White	12000 (44%)	8960 (33%)	8500 (31%)	8720 (32%)	7270 (27%)
Asian	280 (46%)	170 (28%)	150 (24%)	200 (33%)	160 (27%)
Black	130 (39%)	90 (27%)	80 (26%)	80 (25%)	130 (42%)
Chinese or Mixed or Other	160 (43%)	130 (35%)	110 (30%)	80 (23%)	100 (28%)
Missing	1180 (23%)	1310 (26%)	1020 (20%)	500 (10%)	880 (17%)

	Statins	Urate lowering therapy	ACE inhibitors	Proton pump inhibitors	Calcium channel blockers
**IMD Quintile**					
Q1 (least deprived)	3320 (38%)	2620 (30%)	2400 (27%)	2140 (25%)	2120 (24%)
Q2	2990 (40%)	2370 (32%)	2080 (28%)	2020 (27%)	1840 (25%)
Q3	3070 (42%)	2320 (32%)	2190 (30%)	2150 (29%)	1890 (26%)
Q4	2380 (42%)	1880 (33%)	1800 (32%)	1790 (32%)	1490 (26%)
Q5 (most deprived)	1980 (45%)	1440 (33%)	1380 (31%)	1480 (34%)	1200 (27%)

Table 9b: Top 10 drugs class (6 to 10) for index condition 'Gout' (Index Condition Date: <= 30/11/2015) - age, sex, ethnicity, and IMD quintile distributions. Note: For disclosure control, all numbers presented are rounded to 10, percentages are rounded to discrete numbers and percentages presented are row percentages. So totals will not add exactly.

	Cardiovascular Beta blockers	Non-opioid analgesics	Antiplatelets	Opioid analgesics	Angiotensin receptor blockers
**All**					
All	8040 (24%)	7040 (21%)	7020 (21%)	5540 (17%)	4750 (14%)
**Age**					
<25	0 (0%)	0 (6%)	0 (0%)	0 (4%)	0 (0%)
25-34	10 (3%)	20 (5%)	0 (1%)	30 (6%)	0 (0%)
35-44	100 (5%)	130 (6%)	40 (2%)	160 (8%)	40 (2%)
45-54	400 (8%)	410 (8%)	240 (5%)	470 (10%)	280 (6%)
55-64	1170 (17%)	1020 (15%)	880 (13%)	1060 (15%)	790 (11%)
65-74	2510 (29%)	1910 (22%)	2180 (25%)	1580 (18%)	1560 (18%)
75-84	2660 (37%)	2340 (32%)	2490 (35%)	1570 (22%)	1510 (21%)
>=85	1190 (38%)	1210 (38%)	1200 (38%)	680 (22%)	570 (18%)
**Sex**					
Men	5992 (23%)	4540 (17%)	5475 (21%)	3624 (14%)	3369 (13%)
Women	2050 (29%)	2503 (36%)	1550 (22%)	1920 (27%)	1380 (20%)
**Ethnicity**					

	Cardiovascular Beta blockers	Non-opioid analgesics	Antiplatelets	Opioid analgesics	Angiotensin receptor blockers
White	7310 (27%)	6460 (24%)	6460 (24%)	5100 (19%)	4140 (15%)
Asian	130 (21%)	170 (27%)	160 (26%)	110 (17%)	110 (18%)
Black	70 (23%)	80 (26%)	60 (20%)	60 (19%)	50 (17%)
Chinese or Mixed or Other	80 (22%)	70 (19%)	80 (22%)	40 (12%)	50 (14%)
Missing	450 (9%)	270 (5%)	260 (5%)	240 (5%)	400 (8%)
**IMD Quintile**					
Q1 (least deprived)	1920 (22%)	1270 (15%)	1600 (18%)	970 (11%)	1300 (15%)
Q2	1750 (24%)	1400 (19%)	1450 (20%)	1100 (15%)	1130 (15%)
Q3	1790 (25%)	1590 (22%)	1610 (22%)	1180 (16%)	1030 (14%)
Q4	1480 (26%)	1460 (26%)	1280 (23%)	1170 (21%)	760 (13%)
Q5 (most deprived)	1100 (25%)	1330 (30%)	1080 (25%)	1130 (26%)	520 (12%)

Drugs Name (Top 10)

Table 10a: Top 10 drugs name (1 to 5) for index condition 'Gout' (Index Condition Date: <= 30/11/2015) - age, sex, ethnicity, and IMD quintile distributions. Note: For disclosure control, all numbers presented are rounded to 10, percentages are rounded to discrete numbers and percentages presented are row percentages. So totals will not add exactly.

	1001 Allopurinol	0407 Paracetamol	0212 Simvastatin	0103 Omeprazole	0212 Atorvastatin
**All**					
All	10400 (31%)	6770 (20%)	6740 (20%)	6080 (18%)	5980 (18%)
**Age**					
<25	10 (12%)	0 (6%)	0 (0%)	0 (6%)	0 (0%)
25-34	70 (15%)	20 (5%)	0 (1%)	30 (6%)	10 (1%)
35-44	440 (22%)	130 (6%)	40 (2%)	170 (9%)	70 (4%)
45-54	1300 (27%)	400 (8%)	340 (7%)	550 (11%)	420 (9%)
55-64	2140 (31%)	980 (14%)	1070 (15%)	1110 (16%)	1200 (17%)
65-74	3010 (34%)	1820 (21%)	2260 (26%)	1780 (20%)	2100 (24%)
75-84	2500 (35%)	2240 (31%)	2140 (30%)	1690 (23%)	1640 (23%)
>=85	950 (30%)	1160 (37%)	880 (28%)	750 (24%)	530 (17%)
**Sex**					
Men	8730 (33%)	4320 (16%)	5250 (20%)	4460 (17%)	4760 (18%)
Women	1680 (24%)	2440 (35%)	1490 (21%)	1610 (23%)	1220 (17%)
**Ethnicity**					
White	8730 (32%)	6190 (23%)	5880 (22%)	5500 (20%)	5220 (19%)
Asian	170 (27%)	160 (27%)	110 (18%)	110 (19%)	140 (24%)
Black	80 (27%)	80 (26%)	60 (17%)	50 (14%)	60 (20%)
Chinese or Mixed or Other	120 (34%)	60 (18%)	80 (21%)	60 (15%)	70 (18%)
Missing	1290 (25%)	260 (5%)	630 (12%)	360 (7%)	480 (10%)
**IMD Quintile**					
Q1 (least deprived)	2570 (30%)	1210 (14%)	1610 (18%)	1370 (16%)	1450 (17%)
Q2	2320 (31%)	1340 (18%)	1460 (20%)	1300 (18%)	1270 (17%)
Q3	2260 (31%)	1520 (21%)	1450 (20%)	1400 (19%)	1380 (19%)

	1001 Allopurinol	0407 Paracetamol	0212 Simvastatin	0103 Omeprazole	0212 Atorvastatin
Q4	1840 (32%)	1410 (25%)	1200 (21%)	1120 (20%)	1040 (18%)
Q5 (most deprived)	1400 (32%)	1280 (29%)	1030 (23%)	890 (20%)	830 (19%)

Table 10b: Top 10 drugs name (6 to 10) for index condition 'Gout' (Index Condition Date: <= 30/11/2015) - age, sex, ethnicity, and IMD quintile distributions. Note: For disclosure control, all numbers presented are rounded to 10, percentages are rounded to discrete numbers and percentages presented are row percentages. So totals will not add exactly.

	0209 Aspirin	0206 Amlodipine	0205 Ramipril	0204 Bisoprolol	0601 Metformin
**All**					
All	5820 (17%)	5480 (16%)	5370 (16%)	4420 (13%)	3700 (11%)
**Age**					
<25	0 (0%)	0 (0%)	0 (0%)	0 (0%)	0 (0%)
25-34	0 (0%)	0 (1%)	10 (2%)	0 (1%)	0 (1%)
35-44	30 (2%)	70 (4%)	120 (6%)	40 (2%)	60 (3%)
45-54	200 (4%)	410 (8%)	510 (10%)	210 (4%)	340 (7%)
55-64	750 (11%)	1160 (17%)	1110 (16%)	600 (9%)	810 (12%)
65-74	1830 (21%)	1840 (21%)	1650 (19%)	1310 (15%)	1280 (14%)
75-84	2050 (28%)	1400 (19%)	1410 (20%)	1550 (21%)	950 (13%)
>=85	950 (30%)	590 (19%)	560 (18%)	710 (22%)	250 (8%)
**Sex**					
Men	4580 (17%)	4490 (17%)	4350 (16%)	3360 (13%)	2850 (11%)
Women	1250 (18%)	1000 (14%)	1020 (14%)	1060 (15%)	850 (12%)
**Ethnicity**					
White	5330 (20%)	4600 (17%)	4650 (17%)	4120 (15%)	3090 (11%)
Asian	140 (22%)	120 (19%)	90 (15%)	80 (13%)	150 (24%)
Black	50 (17%)	80 (27%)	50 (14%)	40 (11%)	60 (17%)
Chinese or Mixed or Other	70 (19%)	60 (16%)	60 (18%)	40 (11%)	60 (15%)
Missing	240 (5%)	620 (12%)	520 (10%)	140 (3%)	360 (7%)
**IMD Quintile**					
Q1 (least deprived)	1340 (15%)	1380 (16%)	1320 (15%)	1080 (12%)	770 (9%)
Q2	1180 (16%)	1160 (16%)	1100 (15%)	970 (13%)	800 (11%)
Q3	1340 (18%)	1210 (17%)	1180 (16%)	980 (13%)	850 (12%)
Q4	1070 (19%)	950 (17%)	970 (17%)	780 (14%)	700 (12%)
Q5 (most deprived)	900 (21%)	790 (18%)	790 (18%)	610 (14%)	590 (13%)

Drugs (User Selected)

Drugs Class (User Selected)

Table 11 : User selected drug classes (1 to 4) for index condition 'params$indexConditionName' (Index Condition Date: <= 30/11/2015) - age, sex, ethnicity, and IMD quintile distributions. Note: For disclosure control, all numbers presented are rounded to 10, percentages are rounded to discrete numbers, and percentages presented are row percentages and so totals will not add exactly.

	Angiotensin receptor blockers	Oral anticoagulants	Statins	Thiazide diuretics
**All**				
All	4750 (14%)	3380 (10%)	13740 (41%)	3050 (9%)
**Age**				
<25	0 (0%)	0 (0%)	0 (0%)	0 (0%)
25-34	0 (0%)	0 (1%)	10 (3%)	NA (NA%)
35-44	40 (2%)	10 (1%)	120 (6%)	20 (1%)
45-54	280 (6%)	90 (2%)	820 (17%)	150 (3%)
55-64	790 (11%)	270 (4%)	2430 (35%)	520 (8%)
65-74	1560 (18%)	920 (10%)	4740 (54%)	990 (11%)
75-84	1510 (21%)	1420 (20%)	4120 (57%)	1000 (14%)
>=85	570 (18%)	660 (21%)	1500 (48%)	360 (11%)
**Sex**				
Men	3369 (13%)	2593 (10%)	10812 (41%)	2059 (8%)
Women	1380 (20%)	791 (11%)	2931 (42%)	989 (14%)
**Ethnicity**				
White	4140 (15%)	3250 (12%)	12000 (44%)	2610 (10%)
Asian	110 (18%)	30 (4%)	280 (46%)	50 (9%)
Black	50 (17%)	20 (7%)	130 (39%)	50 (15%)
Chinese or Mixed or other	50 (14%)	20 (6%)	160 (43%)	30 (8%)
Missing	400 (8%)	60 (1%)	1180 (23%)	310 (6%)
**IMD Quintile**				

	Angiotensin receptor blockers	Oral anticoagulants	Statins	Thiazide diuretics
Q1 (least deprived)	1300 (15%)	870 (10%)	3320 (38%)	780 (9%)
Q2	1130 (15%)	770 (10%)	2990 (40%)	640 (9%)
Q3	1030 (14%)	750 (10%)	3070 (42%)	700 (10%)
Q4	760 (13%)	570 (10%)	2380 (42%)	530 (9%)
Q5 (most deprived)	520 (12%)	420 (10%)	1980 (45%)	400 (9%)

Outcome - Deaths

Table 12: Number of Deaths for Index Condition 'Gout' (Index Condition Date: <= 30/11/2015) - with follow up for 3 years (30/11/2018). Note: For disclosure control, all numbers presented are rounded to 10, percentages are rounded to discrete numbers, and percentages presented are perecentages of total population of index condition cohort and so totals will not add exactly.

Death (Over years)	No of Deaths (%)
Upto one year or less	1140 (3%)
Upto two year or less	2240 (7%)
Upto three year or less	3400 (10%)

Outcome - Death Rate

Table 13: Death rate for Index Condition 'Gout' (Index Condition Date: <= 30/11/2015) - with follow up for 3 years (30/11/2018). Note: For disclosure control, all numbers presented are rounded to two decimal places.

Death (Over years)	Death Rate
Upto one year or less	3.51
Upto two year or less	3.53
Upto three year or less	3.66

Outcome - Hospital Admissions

Table 14: Hospital admissions for Index Condition 'Gout' (Index Condition Date: <= 30/11/2015) - with follow up for 3 years (30/11/2018). Note: For disclosure control, all numbers presented are rounded to 10, percentages are rounded to discrete numbers, and percentages presented are perecentages of total population of index condition cohort and so totals will not add exactly.

Hospital Admission (Over years)	Admission No (%)
Upto one year or less	990 (3%)
Upto two year or less	2130 (6%)
Upto three year or less	3380 (10%)

Outcome - Hospital Admission Rate

Table 15: Hospital admission rate for Index Condition 'Gout' (Index Condition Date: <= 30/11/2015) - with follow up for 3 years (30/11/2018). Note: For disclosure control, all numbers presented are rounded to two decimal places.

Hospital Admission (Over years)	Admission Rate
Upto one year or less	3

Hospital Admission (Over years)	Admission Rate
Upto two year or less	3.29
Upto three year or less	3.53
