# Supplementary material for: A Digital Tool for Clinical Evidence–Driven Guideline Development by Studying Properties of Trial Eligible and Ineligible Populations: Development and Usability Study
Source: J Med Internet Res. 2025 Jan 16;27:e52385. doi: 10.2196/52385 (PMC11783027; doi:10.2196/52385)
Supplement: Multimedia Appendix 5 [file jmir_v27i1e52385_app5.pdf]

Trial Analysis Report - Eligible vs Ineligible Populations

Shahzad Mumtaz

25-07-2023

Trial Analysis for Gout

This report focuses on the clinical trial comparison for trial eligible and ineligible populations from the Clinical Primary Care Data (CPRD)-Gold data. Following is the summary statistics.

- Total number of patients 33480
- Index condition date 11/30/2015 (All patients registered on or before the specified date with Gout)

Inclusion Criteria

- Sex - Men, Women
- Ethnicity - White, Asian, Black, Chinese or Mixed or Other, Missing
- Age - greater than or equal to 18

Exclusion Criteria

- Comorbidity - End Stage Renal, Disease Alcohol Misuse, Liver Failure
- Drug - 0202 Bendroflumethiazide, 0202 Hydrochlorothiazide, 0202 Cyclopenthiazide, 0202 Indapamide, 0407 Aspirin, 0105 Mesalazine, 0105 Sulfasalazine

Analysis Tables for Gout

The report presents the following tables in terms of trial eligible/ineligible populations based on the clinical trial criteria:

- Trial Analysis Summary
- Demography infomration
- electronic Frailty Index (eFI) distribution
- Charlson score distribution
- Comorbidities (Top 10).
  - Comorbidities Body System (Top 10)
  - Comorbidities Condition Group (Top 10)
  - Comorbidities Individual Condition (Top 10)
- Comorbidities (User Selected).
  - Comorbidities Condition Group (User Selected)
- Drugs (Top 10).
  - Drug Chapter (Top 10)
  - Drug Class (Top 10)
  - Drug Name (Top 10)
- Drugs (User Selected).
  - Drug Class (User Selected)
- Outcome
  - Deaths
  - Death Rate
  - Hospital Admissions
  - Hospital Admission Rate

Trial Results Summary

Table 1: Trial analysis summary - eligible vs ineligible populations. Note: For disclosure control, all numbers presented are rounded to 10 and percentages are rounded to discrete numbers. So totals will not add exactly.

All No(%)	Eligible No(%)	Ineligible No(%)
33480 (100%)	27080 (81%)	6400 (19%)

Demography - Eligible/Ineligible

Table 2: Demography Analysis (age and sex distributions) - eligible vs ineligible populations. Note: For disclosure control, all numbers presented are rounded to 10, percentages are rounded to discrete numbers, and are column percentages for column titled 'All No' and row percentages for columns titled 'Eligible No' and 'Inelgible No' and and so totals will not add exactly.

Criteria	All No(%)	Eligible No(%)	Ineligible No(%)
**All**			
All	33480 (100%)	27080 (81%)	6400 (19%)
**Sex**			
Men	26440 (79%)	21520 (81%)	4920 (19%)
Women	7040 (21%)	5560 (79%)	1480 (21%)
**Age**			
<25	50 (0%)	40 (85%)	10 (15%)
25-34	470 (1%)	420 (91%)	40 (9%)
35-44	1960 (6%)	1740 (89%)	220 (11%)
45-54	4900 (15%)	4220 (86%)	680 (14%)
55-64	6930 (21%)	5580 (81%)	1350 (19%)
65-74	8800 (26%)	6850 (78%)	1960 (22%)
75-84	7210 (22%)	5610 (78%)	1600 (22%)
>=85	3160 (9%)	2610 (83%)	550 (17%)

electronic Frailty Index (eFI) - Eligible/Ineligible

Table 3: electronic Frailty Index (eFI) - eligible vs ineligible populations. Note: For disclosure control, all numbers presented are rounded to 10 and percentages are rounded to discrete numbers and percentages are computed based on totals of eligible and ineligible respectively. So totals will not add exactly.

eFI Category	Eligible No (%)	Ineligible No (%)
Fit	12020 (44%)	1630 (25%)
Mild Frailty	7850 (29%)	2370 (37%)
Moderate Frailty	4620 (17%)	1590 (25%)
Severe Frailty	2600 (10%)	810 (13%)

Charlson Comorbidities Score - Eligible/Ineligible

Table 4: Charlson comorbidites score - eligible vs ineligible populations. Note: For disclosure control, all numbers presented are rounded to 10 and percentages are rounded to discrete numbers and percentages are computed based on totals of eligible and ineligible respectively. So totals will not add exactly.

Charlson Score	Eligible No (%)	Ineligible No (%)
Score 0	9990 (37%)	1510 (24%)
Score 1	4930 (18%)	1140 (18%)
Score 2	4400 (16%)	1190 (19%)
Score 3+	7760 (29%)	2560 (40%)

Comorbidities (Top 10)

Body System Comorbidities (Top 10) - Eligible/Ineligible

Table 5: Top 10 comorbidities - Body system comorbidites for index condition 'Gout' (Index Condition Date: <= 30/11/2015) - eligible vs ineligible populations. Note: For disclosure control, all numbers presented are rounded to 10 and percentages are rounded to discrete numbers and percentages are computed based on totals of eligible and ineligible respectively. So totals will not add exactly.

Condition	Eligible No (%)	Ineligible No (%)
Diseases of the Circulatory System	17230 (64%)	5500 (86%)
Musculoskeletal conditions	10830 (40%)	3100 (48%)
Diseases of the Genitourinary system	10160 (38%)	2980 (47%)
Diseases of the Digestive System	9710 (36%)	3110 (49%)
Diseases of the Endocrine System	9220 (34%)	2760 (43%)
Mental Health Disorders	7710 (28%)	3730 (58%)
Diseases of the Respiratory System	6190 (23%)	1800 (28%)
Cancers	4770 (18%)	1340 (21%)
Neurological conditions	2190 (8%)	800 (12%)
Skin conditions	1720 (6%)	480 (7%)

Condition Group Comorbidities (Top 10) - Eligible/Ineligible

Table 6: Top 10 comorbidities - Condition group comorbidites for index condition 'Gout' (Index Condition Date: <= 30/11/2015) - eligible vs ineligible populations. Note: For disclosure control, all numbers presented are rounded to 10 and percentages are rounded to discrete numbers and percentages are computed based on totals of eligible and ineligible respectively. So totals will not add exactly.

Condition	Eligible No (%)	Ineligible No (%)
Hypertension	15320 (57%)	5230 (82%)
Ulcer and upper GI acid conditions	9330 (34%)	2780 (43%)
Osteoarthritis	8980 (33%)	2550 (40%)
Chronic lung disease	5600 (21%)	1660 (26%)
Depression	5280 (20%)	1760 (28%)
Coronary heart disease	5370 (20%)	1620 (25%)
Diabetes mellitus	5040 (19%)	1610 (25%)
Erectile Dysfunction	4610 (17%)	1420 (22%)
Obesity	4460 (16%)	1330 (21%)
Solid organ cancer - primary	4430 (16%)	1260 (20%)

Individual Condition Comorbidities (Top 10) - Eligible/Ineligible

Table 7: Top 10 comborbidities - Individual condition comorbidites for index condition 'Gout' (Index Condition Date: <= 30/11/2015) - eligible vs ineligible populations. Note: For disclosure control, all numbers presented are rounded to 10 and percentages are rounded to discrete numbers and percentages are computed based on totals of eligible and ineligible respectively. So totals will not add exactly.

Condition	Eligible No (%)	Ineligible No (%)
Hypertension	15320 (57%)	5230 (82%)
Osteoarthritis	8980 (33%)	2550 (40%)
Depression	5280 (20%)	1760 (28%)
Type 2 Diabetes Mellitus	4920 (18%)	1540 (24%)
Coronary Heart Disease (not otherwise specified)	4690 (17%)	1400 (22%)
Erectile Dysfunction	4610 (17%)	1420 (22%)
Gastro-oesophageal Reflux Disease	4650 (17%)	1300 (20%)
Obesity	4460 (16%)	1330 (21%)
Asthma	4260 (16%)	1190 (19%)
Anxiety	3940 (15%)	1330 (21%)

Comorbidities (User Selected)

Condition Group Comorbidities (User selected) - Eligible/Ineligible

Table 8: User selected comorbidities - Condition group comorbidites for index condition 'Gout' (Index Condition Date: <= 30/11/2015) - eligible vs ineligible populations. Note: For disclosure control, all numbers presented are rounded to 10 and percentages are rounded to discrete numbers and percentages are computed based on totals of eligible and ineligible respectively. So totals will not add exactly.

Condition	Eligible No (%)	Ineligible No (%)
Coronary heart disease	5370 (20%)	1620 (25%)
Hypertension	15320 (57%)	5230 (82%)
Ulcer and upper GI acid conditions	9330 (34%)	2780 (43%)
Chronic lung disease	5600 (21%)	1660 (26%)

Drugs (Top 10)

Drugs Chapter (Top 10) - Eligible/Ineligible

Table 9: Top 10 drugs chapter for index condition 'Gout' (Index Condition Date: <= 30/11/2015) - eligible vs ineligible populations. Note: For disclosure control, all numbers presented are rounded to 10 and percentages are rounded to discrete numbers and percentages are computed based on totals of eligible and ineligible respectively. So totals will not add exactly.

Condition	Eligible No (%)	Ineligible No (%)
02 Cardiovascular System	16900 (62%)	5400 (84%)
10 Musculoskeletal and Joint Disease	11790 (44%)	3140 (49%)
01 Gastro-Intestinal System	9010 (33%)	2970 (46%)
04 Central Nervous System	8720 (32%)	3000 (47%)
06 Endocrine System	6900 (25%)	2080 (32%)
03 Resipiratory System	3940 (15%)	1280 (20%)
05 Infections	3670 (14%)	1040 (16%)
09 Nutrition and Blood	3300 (12%)	1400 (22%)
07 Obsetrics, Gynaecology and Urinary-Tract Disorders	2960 (11%)	830 (13%)
11 Eye	2350 (9%)	610 (10%)

Drugs Class (Top 10) - Eligible/Ineligible

Table 10: Top 10 drug classes for index condition 'Gout' (Index Condition Date: <= 30/11/2015) - eligible vs ineligible populations. Note: For disclosure control, all numbers presented are rounded to 10 and percentages are rounded to discrete numbers and percentages are computed based on totals of eligible and ineligible respectively. So totals will not add exactly.

Condition	Eligible No (%)	Ineligible No (%)
Statins	10400 (38%)	3340 (52%)
Urate lowering therapy	8390 (31%)	2250 (35%)
ACE inhibitors	7420 (27%)	2430 (38%)
Proton pump inhibitors	7280 (27%)	2310 (36%)
Calcium channel blockers	6260 (23%)	2290 (36%)
Cardiovascular Beta blockers	6020 (22%)	2020 (32%)
Non-opioid analgesics	5120 (19%)	1930 (30%)
Antiplatelets	5140 (19%)	1880 (29%)
Opioid analgesics	4140 (15%)	1410 (22%)
Angiotensin receptor blockers	3450 (13%)	1300 (20%)

Drugs Name (Top 10) - Eligible/Ineligible

Table 11: Top 10 drug names for index condition 'Gout' (Index Condition Date: <= 30/11/2015) - eligible vs ineligible populations. Note: For disclosure control, all numbers presented are rounded to 10 and percentages are rounded to discrete numbers and percentages are computed based on totals of eligible and ineligible respectively. So totals will not add exactly.

Condition	Eligible No (%)	Ineligible No (%)
1001 Allopurinol	8200 (30%)	2200 (34%)
0407 Paracetamol	5100 (19%)	1660 (26%)
0212 Simvastatin	5130 (19%)	1620 (25%)
0103 Omeprazole	4620 (17%)	1460 (23%)
0212 Atorvastatin	4500 (17%)	1480 (23%)
0209 Aspirin	4210 (16%)	1610 (25%)
0206 Amlodipine	4060 (15%)	1420 (22%)
0205 Ramipril	4100 (15%)	1260 (20%)
0204 Bisoprolol	3380 (12%)	1040 (16%)
0601 Metformin	2830 (10%)	870 (14%)

Drugs (User Selected)

Drugs Class (User Selected) - Eligible/Ineligible

Table 12: User selected drug classes for index condition 'Gout' (Index Condition Date: <= 30/11/2015) - eligible vs ineligible populations. Note: For disclosure control, all numbers presented are rounded to 10 and percentages are rounded to discrete numbers and percentages are computed based on totals of eligible and ineligible respectively. So totals will not add exactly.

Condition	Eligible No (%)	Ineligible No (%)
Angiotensin receptor blockers	3450 (13%)	1300 (20%)
Oral anticoagulants	2660 (10%)	720 (11%)
Statins	10400 (38%)	3340 (52%)
Thiazide diuretics	70 (0%)	2980 (47%)

Outcome - Deaths - Eligible/Ineligible

Table 13: Number of Deaths for Index Condition 'Gout' (Index Condition Date: <= 30/11/2015) - with follow up for 3 years (30/11/2018) - eligible vs ineligible populations. Note: For disclosure control, all numbers presented are rounded to 10 and percentages are rounded to discrete numbers and percentages are computed based on totals of eligible and ineligible respectively. So totals will not add exactly.

**Death (Over years)**	**Eligible No (%)**	**Ineligible No (%)**
Upto one year or less	890 (3%)	250 (4%)
Upto two year or less	1720 (6%)	510 (8%)
Upto three year or less	2610 (10%)	790 (12%)

Outcome - Death Rate - Eligible/Ineligible

Table 14: Death rate for Index Condition 'Gout' (Index Condition Date: <= 30/11/2015) - with follow up for 3 years (30/11/2018) - eligible vs ineligible populations. Note: For disclosure control, all numbers presented are rounded to two decimal places.

**Death (Over years)**	**Eligible Rate**	**Ineligible Rate**
Upto one year or less	3.38	4.07
Upto two year or less	3.36	4.26
Upto three year or less	3.47	4.49

Outcome - Hospital Admissions - Eligible/Ineligible

Table 15: Hospital admissions for Index Condition 'Gout' (Index Condition Date: <= 30/11/2015) - with follow up for 3 years (30/11/2018) - eligible vs ineligible populations. Note: For disclosure control, all numbers presented are rounded to 10 and percentages are rounded to discrete numbers and percentages are computed based on totals of eligible and ineligible respectively. So totals will not add exactly.

Hospital Admission (Over years)	Eligible No (%)	Ineligible No (%)
Upto one year or less	760 (3%)	230 (4%)
Upto two year or less	1640 (6%)	500 (8%)
Upto three year or less	2580 (10%)	800 (12%)

Outcome - Hospital Admission Rate - Eligible/Ineligible

Table 16: Hospital admission rate for Index Condition 'Gout' (Index Condition Date: <= 30/11/2015) - with follow up for 3 years (30/11/2018) - eligible vs ineligible populations. Note: For disclosure control, all numbers presented are rounded to discrete numbers. So totals will not add exactly.

Hospital Admission (Over years)	Eligible Admission Rate	Ineligible Admission Rate
Upto one year or less	2.86	3.6
Upto two year or less	3.11	4.02
Upto three year or less	3.33	4.42
